# The monophyly of Susisuchidae (Crocodyliformes) and its phylogenetic placement in Neosuchia

**DOI:** 10.7717/peerj.759

**Published:** 2015-02-12

**Authors:** Alan H. Turner, Adam C. Pritchard

**Affiliations:** Department of Anatomical Sciences, Stony Brook University, Stony Brook, NY, USA

**Keywords:** Crocodyliformes, Neosuchia, Phylogeny, Cretaceous, Systematics, Morphology

## Abstract

Eusuchian crocodyliforms, which include all living crocodylians, have historically been characterized by two anatomical specializations: a ball-in-socket vertebral joint and an extensive secondary hard palate with a pterygoid-bound internal choana. The Early Cretaceous neosuchian clade Susisuchidae is typically regarded as phylogenetically near Eusuchia. The putative susisuchid *Isisfordia duncani* was initially described as a transitional form exhibiting incipient versions of these eusuchian traits. Here we examine aspects of the morphology of *Isisfordia* and comment on the morphology of its putative sister taxon *Susisuchus*. Our reexamination supports the notion of *Isisfordia* possessing transitional vertebral morphology but we present a new interpretation of its palate construction that shows it to be more plesiomorphic than previously thought. The secondary choana of *Isisfordia* is not pterygoid bound. Instead, long palatines expand distally lapping under the pterygoid to form the anterior border of the choana as is common among many advanced neosuchians. Incorporation of these observations into an expanded phylogenetic dataset of neosuchian crocodyliforms results in a new phylogenetic hypothesis for Susisuchidae. *Isisfordia* and *Susisuchus* form a monophyletic Susisuchidae that sits near the base of Neosuchia, and is not the sister taxon of Eusuchia.

## Introduction

The evolution of the modern crocodylian bauplan throughout the Mesozoic Era represents one of the first-recognized and best-understood macroevolutionary transitions in vertebrate history. Huxley (1875) first recognized a series of anatomical transitions between Triassic archosaurs, Jurassic intermediates (dubbed “mesosuchians”) and the modern eusuchian anatomy. Although substantial complexity is now recognized in the evolution of Mesoeucrocodylia (the clade including “mesosuchians” and Crocodylia), the understanding of the underlying transitions remains (e.g., Langston, 1973; Buffetaut, 1982; Brochu, 2003) and new forms have been recognized as filling in gaps in the sequence (e.g., Clark et al., 2004; Pol et al., 2013).

Salisbury et al. (2003) described a crocodyliform from the Lower Cretaceous Crato Formation of Brazil. This taxon, *Susisuchus anatoceps*, exhibits a mixture of derived osteoderm morphology (it has a tetraserial dorsal shield) and plesiomorphic vertebral morphology (the holotype specimen has amphicoelous vertebrae). Detailed comparisons were made with a range of “advanced neosuchians” such as *Theriosuchus pusillus*, the Glen Rose Form, *Brillanceausuchus*, *Bernissartia*, and an undescribed neosuchian from Lower Cretaceous Winton Formation of Queensland, Australia. *Susisuchus* was so distinct from other neosuchians that Salisbury et al. (2003) established a new “family”-level clade Susisuchidae for it. At the time, he suggested that the undescribed neosuchian from Queensland might also be a member of the family.

Salisbury et al. (2006) later described this new crocodyliform, *Isisfordia duncani*. The authors characterized the taxon as an intermediate between “mesosuchians” and eusuchians, and phylogenetic analysis recovered *Isisfordia* as the sister taxon to a clade containing all other eusuchians in the analysis. It did not recover a monophyletic Susisuchidae, finding *Susisuchus* to be the sister taxon of *Isisfordia* + Eusuchia. *Susisuchus anatoceps* is preserved on a slab with no ventral exposure of its palate. Thus little can be said regarding the palate construction for this taxon and it cannot serve to inform the interpretation of the palate of *Isisfordia* or susisuchids as a whole.

The position of *Isisfordia* was supported by a number of morphological features long considered important in the origin of the eusuchian condition (Huxley, 1875; Langston, 1973; Salisbury & Frey, 2001), particularly in the palate and the vertebral centra. The palate was reconstructed with the secondary choana (sensu Witmer, 1995) enclosed by the pterygoid, the anterior margin framed by bilateral, anteroposteriorly narrow plates (ventral laminae sensu Salisbury et al., 2006). This condition was presented as intermediate between the traditional mesosuchian palatal condition (e.g., Huxley, 1875; Langston, 1973), in which the pterygoid forms only the posterior portion of the secondary choana with the palatines framing the opening anteriorly, and the eusuchian condition, in which the pterygoid fully encloses the choana with the anterior margin formed by anteroposteriorly elongated ventral laminae. The scenario suggested a gradual, anteroposterior elongation of the ventral laminae.

Salisbury et al. (2006) also drew attention to the vertebral centra in *Isisfordia*. All of the non-sacral centra were considered to be “weakly procoelous” (Salisbury et al., 2006). The illustrated centra exhibit a slight posterior convexity, a condition intermediate between the weak concavity of a traditional “mesosuchian” (e.g., Huxley, 1875; Salisbury & Frey, 2001) and the strong, hemispherical cotyle in modern crocodylians. The phylogenetic analysis considered procoely a synapomorphy of the clade *Isisfordia* + (*Hylaeochampsa* + Crocodylia). Salisbury & Frey (2001) and Salisbury et al. (2006) considered this transition correlated with the acquisition of a tetraserial osteoderm shield and the concomitant need for a vertebral bracing system.

The position of *Isisfordia duncani* and/or other susisuchids as the sister group to all other eusuchians has substantial implications on hypotheses of early crocodylian biogeography and functional morphology. However, inclusion of the taxon in other phylogenetic analyses has resulted in alternative positions. Pol, Turner & Norell (2009) noted two alternative most parsimonious positions, one equivalent to Salisbury et al. (2006) and another positioning *Isisfordia* as the sister taxon of *Rugosuchus* + *Shamosuchus*. Andrade et al. (2011) recovered *Isisfordia* + *Susisuchus* as a clade (Susisuchidae), with susisuchids being the sister taxon of *Hylaeochampsa* + Crocodylia. As part of a larger project examining large-scale patterns of neosuchian and basal eusuchian phylogeny (Brochu et al., 2011; Turner & Brochu, 2011; Brochu, 2013; Pritchard et al., 2013; Turner, in press), we restudied all available material of *Isisfordia* and incorporated it into a phylogenetic analysis with a broad sampling of Mesozoic neosuchians. Here we present character analysis specific to the anatomy of *Isisfordia duncani*, detail the resulting revisions to scorings in the character matrix, and present an alternate hypothesis for the phylogenetic position of Susisuchidae.

## Material and Methods

### Specimens examined

#### Susisuchus anatoceps

SMNK 3804 PAL (holotype)—Partial articulated skeleton, including the skull, forelimb, and axial column. No pelvis or hind limb is preserved.

#### Isisfordia duncani

QM F36211 (holotype)—Partial articulated skeleton, including the posterior portion of the skull; articulated cervical, dorsal, and caudal regions; left pectoral girdle and forelimb, right scapula and fragmentary coracoid; nearly complete right and left forelimbs; complete dermal shield from the shoulders to the tail. QM F44320 (paratype)—a skull lacking a mandible. QM F44319 (paratype)—partial mandible. QM F34642—partial articulated skeleton, smaller than the holotype skeleton, including most of a snout and braincase; articulated cervical, dorsal, and caudal regions; nearly complete hindlimb and pelvis.

### Comparative sample

Fossil neosuchian material used for comparisons is listed in Table 1. The table lists the taxon name, the collection number of the most informative specimen, and the reference for that taxon. Comparisons for the listed taxa are based on these specimens unless otherwise noted. The terminology for the major crocodyliform clade names Mesoeucrocodylia and Neosuchia follow Clark (in Benton & Clark, 1988). The term “advanced neosuchians” is used here for a group of deeply nested neosuchians more crownward than Goniopholididae and includes such taxa as *Shamosuchus*, *Paralligator*, *Acynodon*, *Allodaposuchus*, *Borealosuchus*, and Crocodylia.

**Table 1:** Fossil neosuchian material used for comparisons.

Species	Specimen number	Reference
*Allodaposuchus subjuniperus*	MPZ 2012/288	Puértolas-Pascual, Canudo & Moreno-Azanza, 2013
*Acynodon iberoccitanus*	ACAP-FX1; ACAP-FX2	Buscalioni, Ortega & Vasse, 1997; Martin, 2007
*Acynodon adriaticus*	MCSNT 57248	Delfino et al., 2008
*Alligator mississippiensis*	FMNH 8201	Brochu, 1992
*Batrachomimus pastosbonensis*	LPRP/USP-0617^a^	Montefeltro et al., 2013
*Crocodylus acutus*	AMNH R-7121	
*Elosuchus cherifiensis*	MNHN SAM 129	Lapparent de Broin, 2002
*Eutretauranosuchus delfsi*	AMNH FARB 570; CMNH 8028	Mook, 1967
Glen Rose Form	MCZ 4453; USNM 22039	Langston, 1973; Langston, 1974
*Goniopholis simus*	NHMUK 41098	Mook, 1942; Clark, 1986; Clark, 1994; Salisbury, 2002
*Pachycheilosuchus trinquei*	SMU 75278 and referred material	Rogers, 2003
*Paluxysuchus newmani*	SMU 76601^a^, 76602^a^	Adams, 2013
*Rhabdognathus*	CNRST-SUNY 190^a^	Brochu et al., 2002
*Rugosuchus nonganensis*	IGV 33^a^	Wu, Cheng & Russell, 2001
*Shamosuchus djadochtaensis*	AMNH FARB 6412; IGM 100/1195	Mook, 1924
*Susisuchus anatoceps*	SMNK 3804 PAL	Salisbury et al., 2003; Figueiredo et al., 2011
*Theriosuchus pusillus*	NHMUK R48328, NHMUK R48330	Owen, 1879; Clark, 1986; Clark, 1994
*Theriosuchus sympleistodon*	FGGUB R.1782	Martin, Rabi & Csiki, 2010; Martin et al., 2014a; Martin et al., 2014b
*Wannchampsus kirpachi*	SMU 76604^a^, 76605^a^	Adams, 2014

**Notes.**

a Denotes specimens not seen firsthand by either of the authors.

### Phylogenetic analysis

#### Taxon sampling

The complete dataset included 101 crocodylomorph taxa plus the outgroup (*Gracilisuchus stipanicicorum*) used to root the phylogenetic trees. The sampling scheme follows that of Turner (in press).

### Dataset and analysis

The dataset used here is identical to that of Turner (in press). Details of the character set are in Appendix S2. Reference specimens and literature consulted for information on the ingroup taxa are available in Appendix S1 and the full dataset, as well as files related to the sensitivity analyses, are available on MorphoBank (O’Leary & Kaufman, 2007) at www.morphobank.org/permalink/?P1200. The phylogenetic dataset was analyzed with equally weighted parsimony using TNT v.1.0 (Goloboff, Farris & Nixon, 2008a; Goloboff, Farris & Nixon, 2008b). A heuristic tree search strategy was conducted performing 10,000 replicates of Wagner trees (using random addition sequences, RAS) followed by TBR branch swapping (holding 10 trees per replicate). The best trees obtained at the end of the replicates were subjected to a final round of TBR branch swapping. Zero-length branches were collapsed if they lacked support under any of the most parsimonious reconstructions (i.e., rule 1 of Coddington & Scharff, 1994).

The character support of the nodes present in the most parsimonious reconstructions was calculated using two different methods. The first technique is the jackknife applied to character resampling (Farris et al., 1996). The second method used is Bremer support (Bremer, 1988; Bremer, 1994), which evaluates node stability/sensitivity by exploring suboptimal tree solutions in order to determine how many additional steps must be allowed in searching for topologies before the hypothesized clade is no longer recovered. The jackknife support analysis was calculated using TNT (Goloboff, Farris & Nixon, 2008a; Goloboff, Farris & Nixon, 2008b). The analysis was performed using 1,000 replicates for which the probability of independent character removal was set to 0.20. Each jackknife replicate was analyzed using a tree search strategy consisting of 10 replicates of RAS followed by TBR branch swapping (saving 10 trees per replicate). The topologies obtained during the jackknife replicates are summarized using GC frequencies (Goloboff et al., 2003). Bremer support was calculated using the BREMER.RUN script provided with TNT.

## Results

### Morphological observations and dataset changes

#### Secondary palate

**Figure 1:**
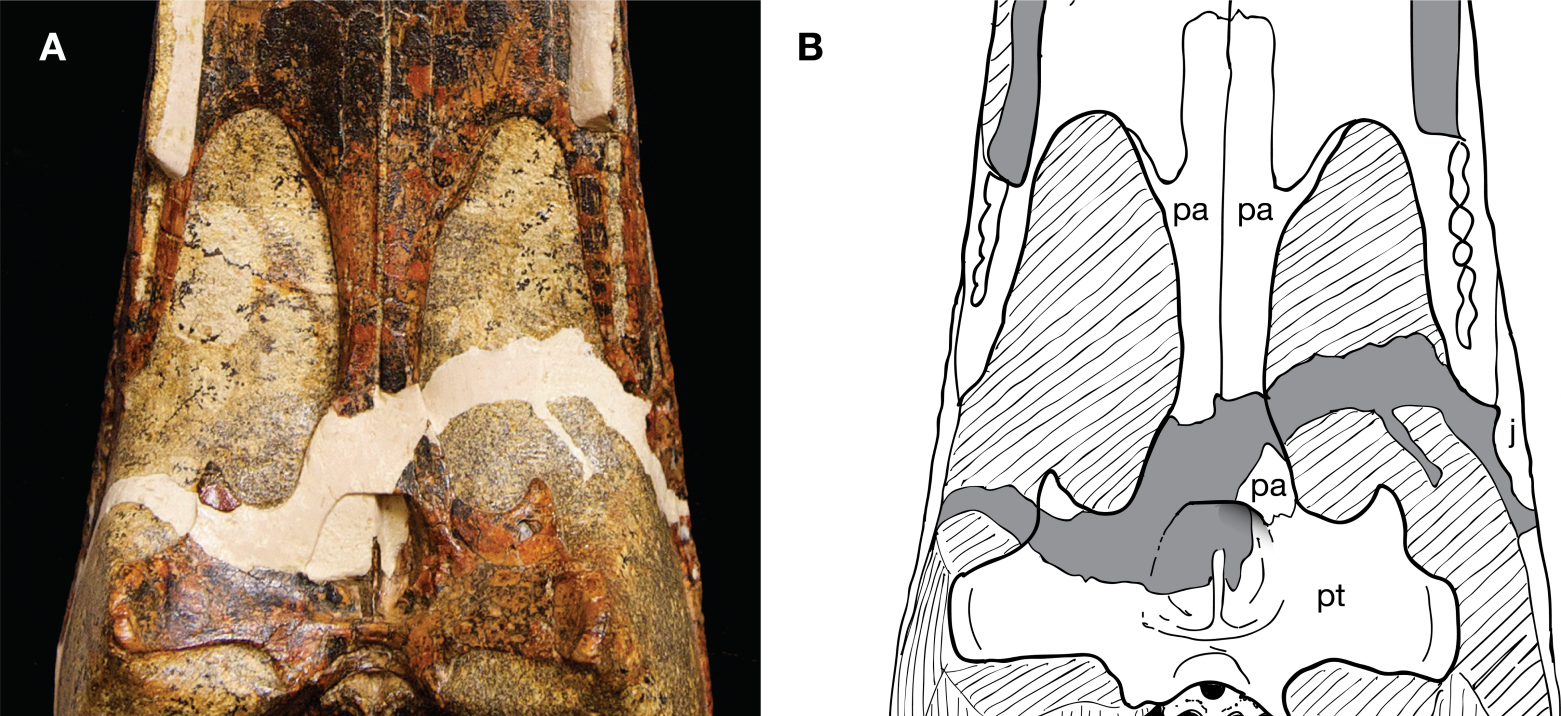
Paratype skull of *Isisfordia duncani* (QM F44320), ventral view of secondary palate. (A) Photograph. (B) Interpretative line drawing of sutures.

The paratype skull of *Isisfordia* (and only specimen of a susisuchid preserving the choana or with an exposed palate) presents a challenge for interpreting the sutural relationships between the pterygoid and palatines. A fracture split the skull diagonally into two pieces, one containing the snout and the other the braincase, posterior portion of the palate, and quadrates. The two pieces were skillfully fit together but the fracture resulted in damage to the nasopharyngeal passage and anterior margin of the pterygoid where it contacts the palatine (Fig. 1). Fortunately, the left pterygoid and palatine are not as extensively damaged as their contralateral pair. Thus, whereas some morphological information is indeed present in its current state, the interpretation of the sutural relationships of the palatine with the pterygoid is a bit more ambiguous than one would like. Further complicating interpretation of sutural relationships is the presence of heavy black mineralization in the form of splotches and veins. The pterygoid has numerous micro-fractures that are also often associated with black mineralization. Lastly, small ridges and grooves frame the lateral margin of the choana, adding further complexity to the region where one would expect the palatine/pterygoid contact to be.

**Figure 2:**
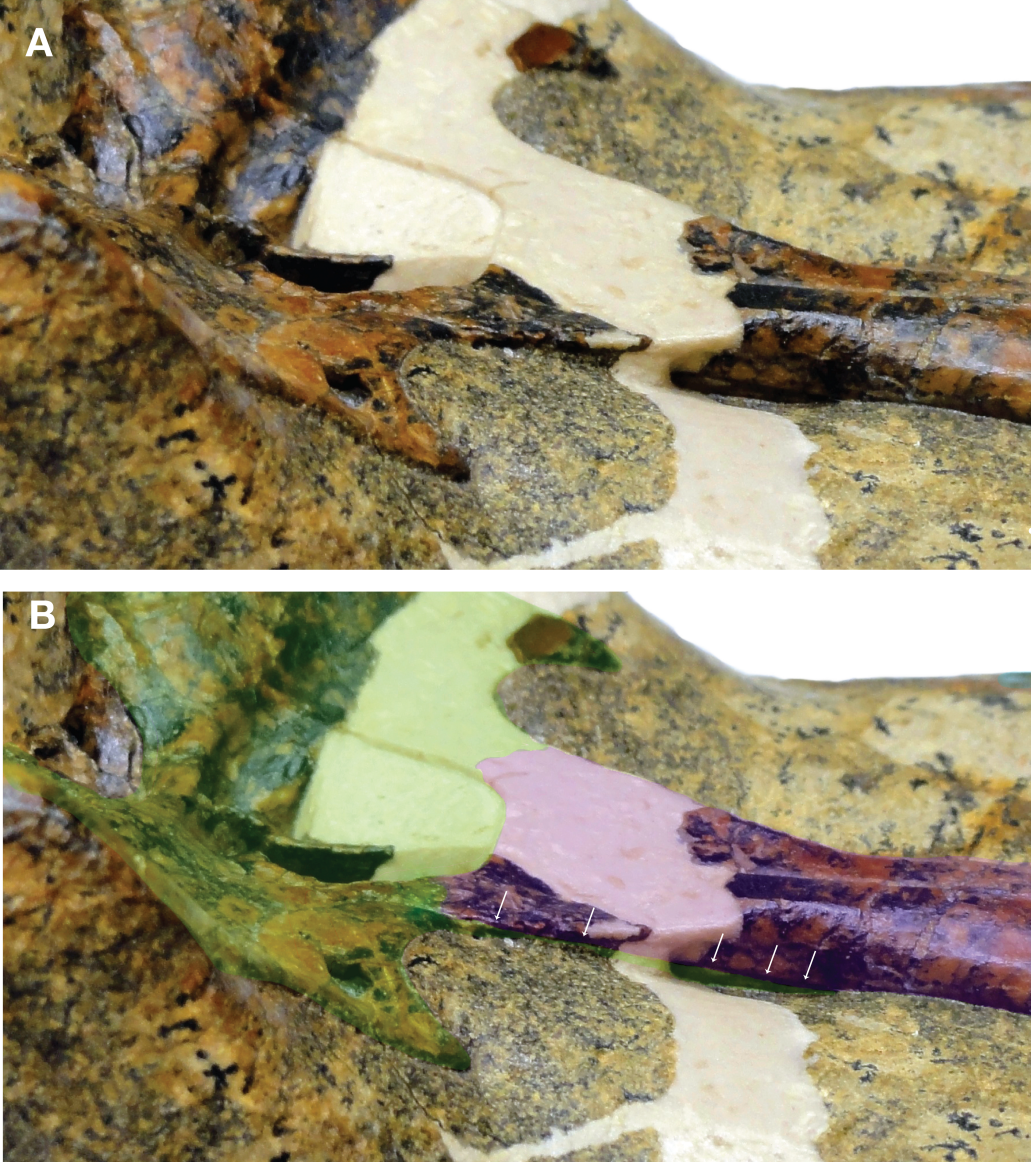
Close-up of QM F44320 illustrating the proposed sutural relationship of the secondary choana (external view). (A) Photograph of skull in left oblique view. (B) Same photograph as in (A) but with bones highlighted to show construction of the secondary choana. Palatines in purple, pterygoid in green. Row of arrows indicates the path of the palatine/pterygoid suture on the dorsal surface of the nasopharyngeal passage.

**Figure 3:**
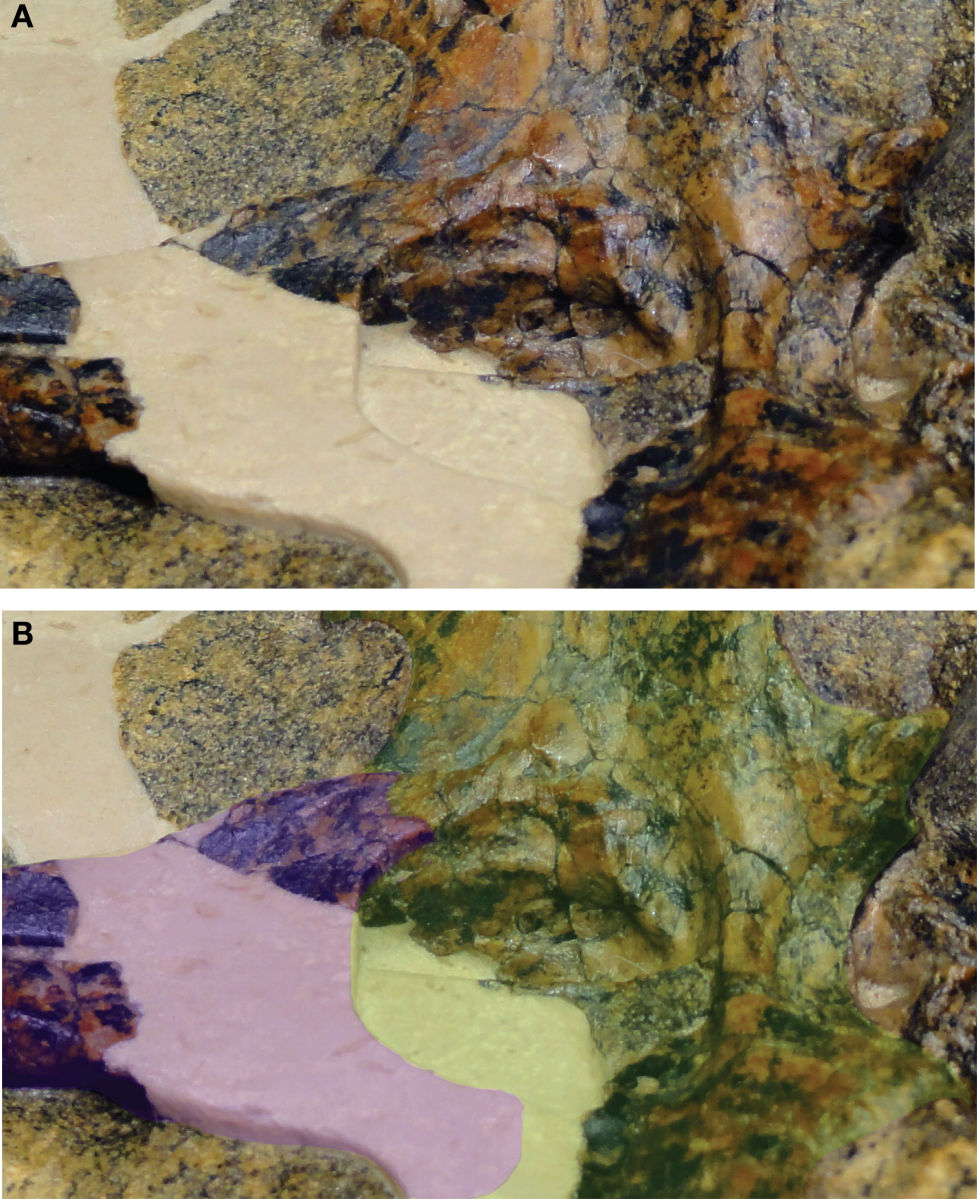
Close-up of QM F44320 illustrating the proposed sutural relationship of the secondary choana (internal view). (A) Photograph of skull in right oblique view. (B) Same photograph as in (A) but with bones highlighted to show construction of the secondary choana. Palatines in purple, pterygoid in green.

Salisbury et al. (2006) reconstructed the palatine-pterygoid contact in an irregular suture slightly anterior to the choana. Thus, *Isisfordia* was considered to have the derived eusuchian condition of an entirely pterygoid-bound choana. The contact identified by Salisbury et al. (2006) as sutural we interpret here as a thin, partially transmitting fracture on the palatine. We interpret a more posterior line, lapping on the pterygoid in ventral view and extending distally before curving back anteriorly to form the anterior margin of the choana, to be the sutural contact between the palatine and pterygoid (Figs. 1–3). Therefore we view *Isisfordia* as lacking the derived pterygoid-bound choana.

A number of lines of evidence support this interpretation. If our interpretation of *Isisfordia* is correct, it suggests that the posterior ends of the palatines expand laterally just anterior to the pterygoid contact (Fig. 1). Similar palatine expansions occur in many other advanced neosuchians (Pol, Turner & Norell, 2009; Turner, in press). This is evident in paralligatorids like *Shamosuchus djadochtaensis*, *Rugosuchus nonganensis*, and *Paralligator gradilifrons*, as well as the putative paralligatorid *Batrachomimus pastosbonensis*. It also appears to be the condition in *Theriosuchus pusillus*. This contrasts with palatines that remain narrow throughout their lengths (e.g., *Elosuchus cherifiensis*, *Eutretauranosuchus delfsi*, *Wannchampsus*, Glen Rose Form). In no eusuchians that possess laterally expanded palatines just anterior to the pterygoid contact (e.g., *Allodaposuchus subjuniperus*, *Allodaposuchus* cf. *precedens*, *Acynodon iberoccitanus*, *Alligator mississippiensis*, *Crocodylus acutus*) does the pterygoid/palatine suture cut across this posterior expansion of the palatines. Instead, the expanded area of the nasopharyngeal passage is always formed by the palatines, with the palatines contacting the pterygoid posterior to the expansion.

In most advanced neosuchians outside of Eusuchia, each palatine is overlapped by the pterygoid in a shallow interdigitating suture before curving back to the midline (e.g., *Goniopholis simus*, *Wannchampsus*, *Bernissartia*, *Paralligator*). This results in the posterior margin of the joined palatines forming a curved or V-shaped anterior margin to the choana. The pterygoid extends anteriorly at a very shallow angle along the dorsal surface of the palatines to frame the dorsolateral portions of the nasopharyngeal passage. We see a similar conformation of palatine and pterygoid in *Isisfordia*. Tracing the lateral margin of the palatine along its expanded posterior end, we see it extend onto the ventral surface of the pterygoid and form a short suture before curving back anteriorly (Figs. 2 and 3). Thus the anterior margin of the choana is palatine-formed and takes a gentle curved shape like that seen in paralligatorids (Turner, in press), *Bernissartia*, and some dyrosaurids (e.g., *Rhabdognathus*) and unlike the V-shape margin of *Theriosuchus pusillus* or the narrow straight margin of *Wannchampsus* (Adams, 2014) or the Glen Rose Form (Langston, 1973; Langston, 1974).

An oblique lateral view of the nasopharyngeal passage on the left side reveals the overlapping contact of the pterygoid on the dorsal surface of the palatine (Fig. 2). Tracing this contact posteriorly, it appears continuous with the line on the pterygoid we have interpreted to be the palatine/pterygoid contact. Thus, it is our view that the choana of *Isisfordia* is not pterygoid-bound but instead shows an intermediate neosuchian condition wherein the palatines contribute extensively to the posteriormost floor of the nasopharyngeal passage, flare laterally at their contact with the pterygoid, but still form a gently curved anterior margin to the choana.

### Vertebrae

Vertebral material has been described from two specimens of *Susisuchus anatoceps* (see Salisbury et al. (2003) and Figueiredo et al. (2011)). The holotype (SMNK 3804 PAL) vertebral series consists of the axial column up to the mid-caudal region. This is preserved on a limestone slab and only exposed in dorsal view. The cervical series is partially obscured by the nuchal osteoderms. The cervical and dorsal vertebrae referred to *Susisuchus anatoceps* by Figueiredo et al. (2011) are three-dimensionally preserved, prepared in articulation, and described in detail.

**Figure 4:**
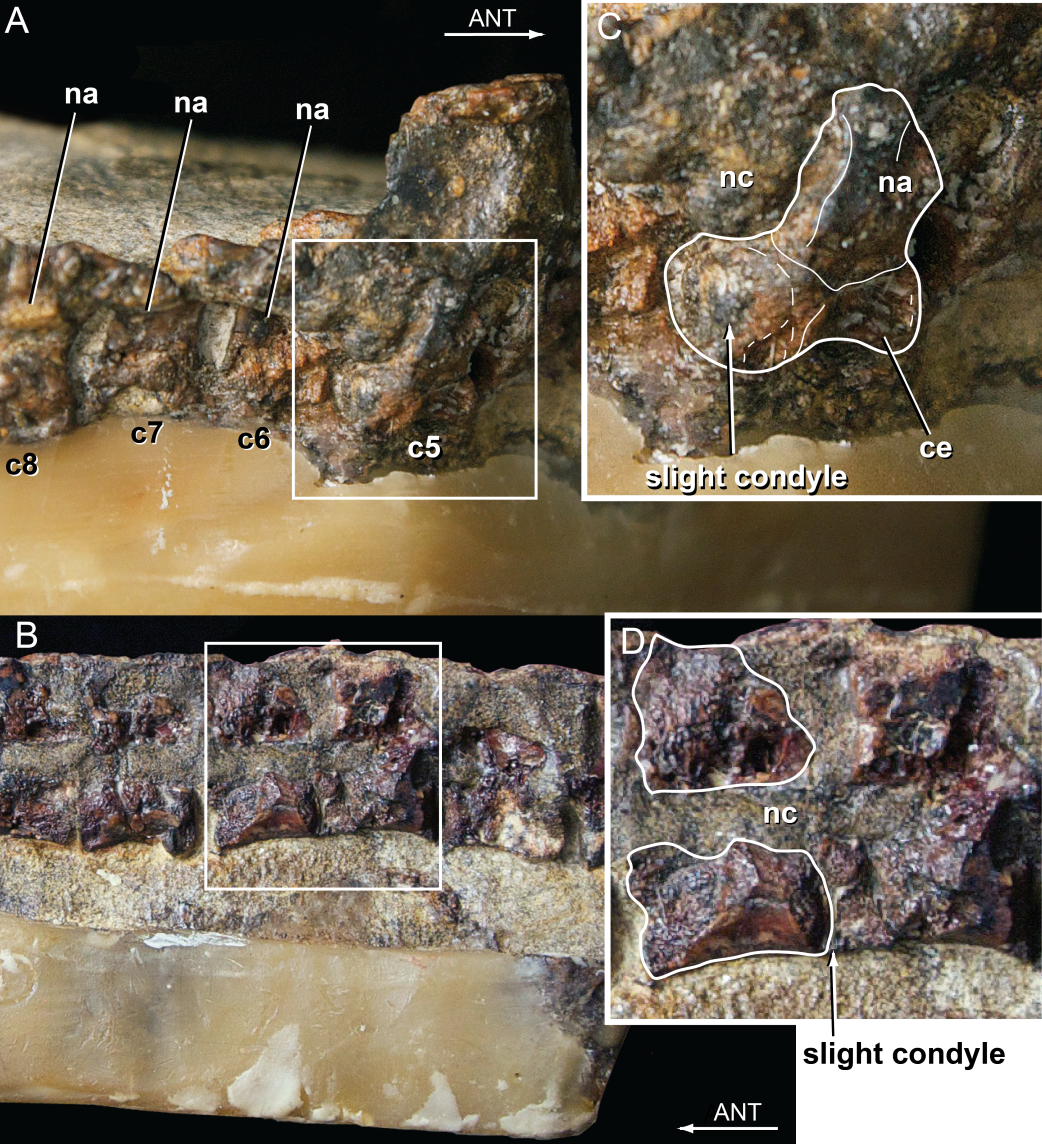
Vertebral anatomy of *Isisfordia duncani*. (A) Mid- to posterior cervical vertebrae of QM F36211 in right lateral view, with body of 5th cervical in posterolateral view. (B) Close-up of 5th cervical highlighting the slight posterior condyle present on the vertebra. (C) Mid-dorsal vertebrae of QM F36211 in right lateral view. (D) Close-up of vertebra showing slight posterior condyle. Abbreviations: ANT, anterior; c, cervical; ce, cervical vertebral body; na, neural arch; nc, neural canal.

**Figure 5:**
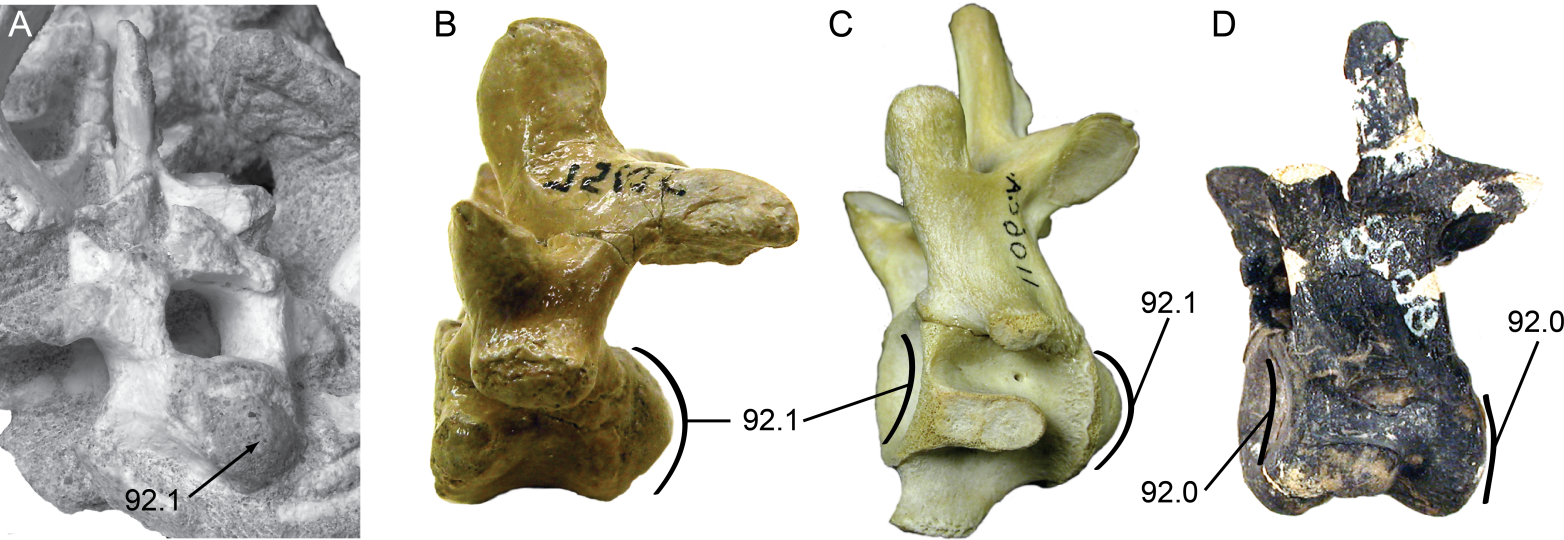
Posterior cervical vertebrae showing the character states for procoely and amphicoely used in the phylogenetic dataset. Anterior is to the left of the page. Labels follow the convention ‘character.character state’. (A) *Shamosuchus djadochtaensis* (IGM 100/1195); (B) *Pachycheilosuchus trinquei* (SMU 75105); (C) *Alligator mississippiensis* (AMNH 1106 C.A.); (D) *Eutretauranosuchus delfsi* (CMNH 8028). (A)–(C) Illustrate the derived condition of strong procoely with a well-developed posterior condyle on the vertebral body. (D) Illustrates the plesiomorphic condition of amphicoely where the anterior cotyle is weakly concave and there is no posterior condyle, just a weakly concave cotyle. Images in (B) and (C) are reversed to aid comparison.

Vertebral material for *Isisfordia duncani* is likewise abundant. The holotype QM F36211 preserves articulated portions of the cervical, dorsal, and caudal regions of the column. The preserved segment of the cervical column in QM F36211 contains the first nine cervical vertebrae and the first dorsal vertebra in articulation (Fig. 4A). None of the vertebrae are complete as a portion of the right half of each has been sheared off. The vertebrae are visible in right lateral view, such that one observes the sheared surface of the elements. Cervicals 6 through 9 (C6–C9) are represented mostly by the neural arch. Vertebral centra are better preserved on C2 through C5. The axial centrum appears to lack a hypapophysis, a condition shared with a specimen referred to *Susisuchus anatoceps* (Figueiredo et al., 2011). A subtle convexity is apparent on the posterior surface of the centra of C2, C3, and especially C5 (Fig. 4B). Further posteriorly, the centra are too poorly preserved to assess this morphology. This subtle convexity is what Salisbury et al. (2006) characterized as “incipient procoely”, and is similar to the weak procoely observed by Figueiredo et al. (2011) in a referred specimen of *Susisuchus anatoceps*. This minor development of procoely is in contrast to the well-developed procoely present in many other advanced neosuchians (e.g., *Shamosuchus*, *Pachycheilosuchus*, *Acynodon adriaticus*) (Fig. 5), and as such appears transitional from the plesiomorphic amphicoelous condition.

The preserved segment of the dorsal region in QM F36211 preserves nine partial dorsal vertebrae in articulation and fragments of a tenth posteriorly (Fig. 4C). As in the cervical region, these have been sheared through vertically, although these are visible in section from a left lateral perspective. In each preserved centrum, a subtle posterior convexity is apparent.

### Phylogenetic analysis

**Figure 6:**
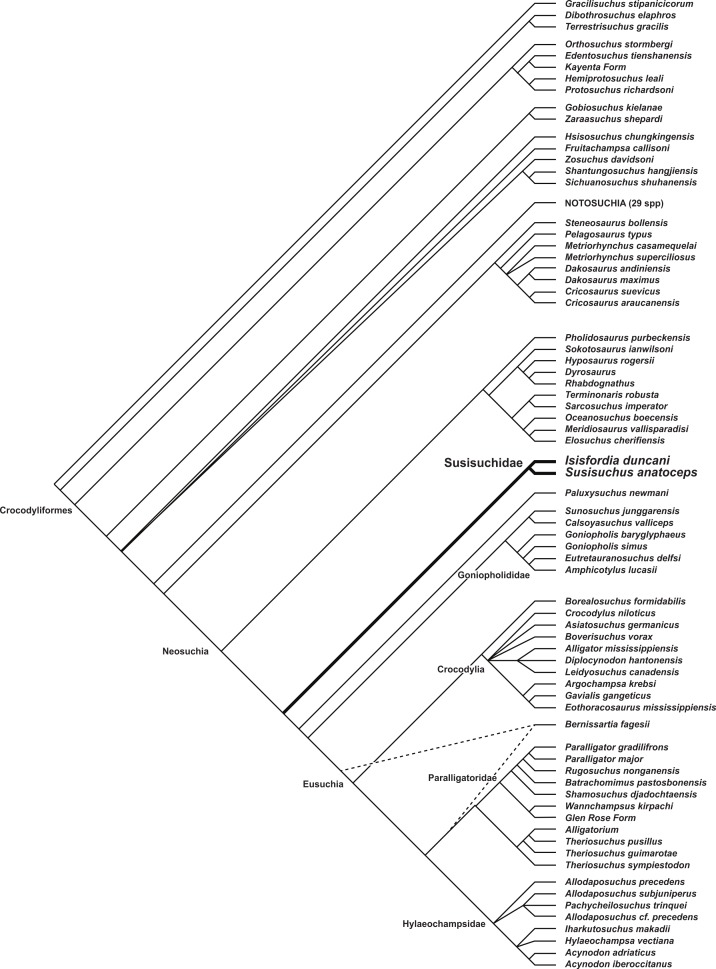
Phylogenetic placement of Susisuchidae. Reduced strict consensus of 108 equally optimal trees recovered from maximum parsimony analysis of 101 ingroup taxa and 318 phenotypic characters. Trees rooted on *Gracilisuchus stipanicicorum*. *Bernissartia fagesii* was excluded during calculation of the strict consensus (but not during the parsimony analysis) due to the conflicting locations it can take in the most parsimonious trees. The two equally optimal positions of *Bernissartia fagesii* are shown with dotted line (length = 1,662; CI = 0.239; RI = 0.700). Susisuchidae node has Bremer value of 4 and a GC value of 57. See Fig. 7D for the strict consensus topology not excluding *Bernissartia* during calculation.

Maximum parsimony analysis recovered 108 optimal trees with a length of 1,662 steps (CI = 0.239, RI = 0.700). A reduced strict consensus of the trees (Fig. 6) unites *Isisfordia* and *Susisuchus* as a monophyletic Susisuchidae. This clade is the secondmost basal divergence within Neosuchia. A pholidosaurid + dyrosaurid clade is at the base of Neosuchia. Susisuchidae is separated from Eusuchia by *Paluxysuchus* and a goniopholidid clade. Eusuchia is comprised of three large clades: Crocodylia, a Paralligatoridae + *Theriosuchus* clade, and a speciose Hylaeochampsidae. The derived position of *Shamosuchus* and other paralligatorids, not recovered in most analyses of mesoeucrocodylian phylogeny, is discussed in detail by Turner (in press). Support metrics are generally low for most neosuchian clades. Susisuchidae has a Bremer value of 4 and GC of 57, representing moderate clade support and some of the higher values among neosuchians. The susisuchid + derived neosuchian node also has a Bremer value of 4 but was not recovered in the jackknife analysis. The discussion that follows will concentrate on character support for the basal position of Susisuchidae and the phylogenetic sensitivity of the clade to the morphological observations made above.

## Discussion

### Phylogenetic position of Susisuchidae

Reanalysis of the phylogenetic position of putative  susisuchids incorporating character state changes based on the observations discussed above results in *Isisfordia* and *Susisuchus* no longer being eusuchians but instead occupying a more basal position within Neosuchia compared to previous analyses (e.g., Salisbury et al., 2006; Pol, Turner & Norell, 2009; Andrade et al., 2011). Six synapomorphies unite *Isisfordia* and *Susisuchus*. Citation of characters from the phylogenetic dataset follows the convention of (character.character state). Characters uniting Susisuchidae include nasals that contribute to the border of the naris (13.0), dorsal osteoderms that lack an anterior articular process (96.0), symmetrical lateral compression of maxillary teeth (140.2), exposure of the supraoccipital on the skull roof (171.1) and the absence of a shallow fossa at the anteromedial corner of the supratemporal fenestra (265.1). One character, the presence of a pear-shaped external naris (309.1) is unique to susisuchids.

A seventh character may also diagnose this clade. Both *Isisfordia* and *Susisuchus anatoceps* each have an extremely large incisive foramen. This morphology is currently represented in the dataset by character 7, which codes for whether the palatal parts of the premaxillae meet behind the incisive foramen. They do not meet in *Isisfordia* due to the massive size of the foramen. In *Susisuchus*, because of preservation, it is uncertain if the massive foramen similarly interrupts the premaxillae posteriorly, although it seems likely that it does.

Susisuchidae is united with neosuchians more derived than the dyrosaurid + pholidosaurid clade based on six synapomorphies. These include changes to the skull like a platyrostral snout (3.3), a cylindrical postorbital bar (26.1), and the jaw joint placed at the level of the occipital condyle (105.0). Additionally, hypapophyses are only present on the cervical vertebrae (91.1) and the posterior half of the axial neural spine is narrow (258.1). Members of this clade of neosuchians are also characterized by a radiale with the proximal end more expanded proximolaterally than proximomedially (117.1).

Susisuchids occupy a basal divergence within Neosuchia due to the absence of a number of features present in more advanced forms. Susisuchids have no variation in maxillary tooth size (79.0), whereas *Paluxysuchus* and the majority of sampled advanced neosuchians have two waves of maxillary tooth enlargement (79.2). This trait is further derived to a single wave of tooth enlargement (79.1) in hylaeochampsids and in most atoposaurids (not *Theriosuchus pusillus*) and *Shamosuchus djadochtaensis*. In dorsal view, susisuchids have a straight lateral margin of the snout (178.0) and thus lack the sinusoidal lateral contour seen in nearly all advanced neosuchians (178.1). Likewise, *Isisfordia* has transversely expanded prefrontal pillars (182.0) whereas all other advanced neosuchians have prefrontal pillars that are transversely expanded in their dorsal part and columnar ventrally (182.1) or longitudinally expanded in their dorsal part (182.3) as in brevirostrine crocodylians. Lastly, susisuchids have a straight ventral margin of the maxilla in lateral view (183.0), whereas all other advanced neosuchians have a sinusoidal central margin of the maxilla in lateral view (183.1—reversed in *Calsoyasuchus*, *Theriosuchus sympiestodon*, *Iharkutosuchus*, *Acynodon*, and *Argochampsa*).

### Sensitivity to interpretation of palate and vertebral morphology

**Figure 7:**
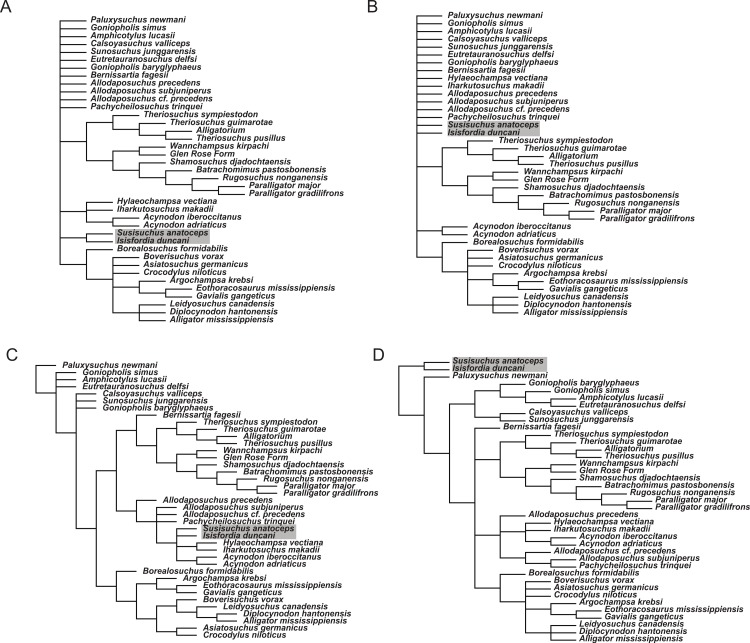
Phylogenetic results of sensitivity analyses exploring alternate character scorings for *Isisfordia duncani*. (A) *Isisfordia duncani* scored for a eusuchian-style palate (43.1) (tree length = 1,663); (B) *Isisfordia duncani* scored for fully-developed procoely (92.1, 93.1) but not for a eusuchian-style palate (43.0) (tree length = 1,664); (C) *Isisfordia duncani* scored for a eusuchian-style palate (43.1) and fully-developed procoely (92.1, 93.1) (tree length = 1,663); (D) *Isisfordia duncani* scored for an alternate character state coding for incipient procoely (tree length = 1,659). All trees depicted are strict consensus topologies of advanced neosuchians in the analysis. TNT files and tree files are available on MorphoBank. Topology in (D) is identical to the strict consensus topology in the primary analysis.

To examine how sensitive the phylogenetic placement of susisuchids  are to the interpretation of the palate and vertebral morphology, alternate scorings for *Isisfordia* were made and phylogenetic trees were estimated from these alternate datasets. Scoring *Isisfordia* as derived for a eusuchian-style palate (43.1) but plesiomorphic for vertebral centrum morphology (92.0; 93.0) results in trees 1,663 steps long (Fig. 7A). This is one step longer than the most parsimonious trees from the primary analysis but, in this case, there is significant reduction in phylogenetic resolution among neosuchians. The clade containing *Theriosuchus* + paralligatoridae is monophyletic and resolved, as is Crocodylia, Susisuchidae, and most of Hylaeochampsidae.

Scoring *Isisfordia* as derived for procoely (92.1; 93.1) but not the palate (43.0) results in trees 1,664 steps long and with even less resolution among neosuchians (Fig. 7B). Only Crocodylia and the *Theriosuchus* + Paralligatoridae clade remain resolved. *Isisfordia* is in a large polytomy at the base of advanced neosuchians.

Scoring of *Isisfordia* as derived for a eusuchian-style palate (43.1) and for procoely (92.1; 93.1) results in trees 1,663 steps long (Fig. 7C). This is one step longer than the most parsimonious trees from the primary analysis. Tree topology among advanced neosuchians is broadly similar to that present in the primary phylogenetic analysis. The trees differ in that Goniopholididae is paraphyletic with respect to the rest of Neosuchia, *Bernissartia* is the sister taxon to the *Theriosuchus* + Paralligatoridae clade, and bizarrely, *Isisfordia* and *Susisuchus* nest within hylaeochampsids but do not always form a susisuchid clade.

Whereas our reinterpretation of the palate construction of *Isisfordia* unquestionably changes how it is scored within our dataset (i.e., it should not be scored as having a pterygoid-bound choana), we do agree with the previous characterization of the vertebral morphology as being “weakly procoelous” or “incipient” in the degree of procoely present. Our primary departure therefore lies in our choice not to homologize the weak procoely of *Isisfordia* and *Susisuchus* (at least MPSC-R1136) with the well-developed procoely in other advanced neosuchians, as has been done by various authors subsequent to its description (Salisbury et al., 2006; Andrade et al., 2011). If the morphological variation present in the vertebrae of Susisuchidae is to be distinguished from the plesiomorphic condition, that it is best done as a separate character state coding for the incipient procoely exhibited by these forms. We explored this option by including an additional incipient procoely state to character 92. Scoring *Isisfordia* and *Susisuchus* for this character state did not change the phylogenetic position of the two taxa or deviate in any other way from the primary analysis topology (Fig. 7D). The only difference was in the number of steps for the most parsimonious tree (in this case 1,659). Figueiredo et al. (2011) scored *Susisuchus* for a similar “semi-procoelous” state in their re-analysis of the phylogenetic position of the taxon based on the dataset of Jouve (2009). They recovered *Susisuchus* outside of a *Shamosuchus* + Crocodylia node, a topology differing from that presented here only in the placement of Goniopholididae.

## Conclusions

Examination of fossil material pertaining to the transitional neosuchian *Isisfordia duncani* resulted in a reinterpretation of the construction of its secondary palate. Coupled with new remains of its close relative *Susisuchus anatoceps*, the vertebrae of susisuchids were considered in a new light. This study resulted in a rescoring of *Isisfordia* into a phylogenetic dataset with a broad sampling of advanced neosuchians (Turner, in press), wherein the secondary choana of *Isisfordia* was not considered pterygoid-bound. Likewise, we chose not to homologize the incipient vertebral procoely of *Isisfordia* and *Susisuchus* with the well-developed derived state (compare Figs. 4 and 5). Combined, these two scoring changes to *Isisfordia* resulted in a phylogeny depicting a more basal divergence within Neosuchia than in previous analyses. Even a more nuanced treatment of the vertebral morphology incorporating an “incipient procoely” state does not overturn this new phylogenetic position.

Given the reinterpretation of the palate of *Isisfordia*, it is not altogether unexpected to see Susisuchidae occupy a more basal position within Neosuchia. It is intriguing that the current character distrubutions support Susisuchidae as more basal than Goniopholididae relative to the crown. Goniopholidids have completely amphicoelous vertebrae and lack the tetraserial vertebral shield present in susisuchids. Relative to susisuchids and advanced neosuchians, the palate of North American goniopholidids appears quite plesiomorphic with palatines that do not meet or do not completely meet at the midline (Pritchard et al., 2013). Three of the four characters that place susisuchids basal to goniopholidids pertain to the shape of the snout and alveolar margin (79.0; 178.0; 183.0). Some recent phylogenetic analyses have placed goniopholidids in a clade with pholidosaurids (Martin & Buffetaut, 2012; Martin et al., 2014a; Martin et al., 2014b); a topology not recovered in our analysis. If this competing hypothesis is correct it could point to poor estimation of the neosuchian root in our analysis, which may explain the basal position of susisuchids relative to goniopholidids. What is certainly true is that additional fossil susisuchid remains and additional character analysis are needed to further address phylogenetic relationships at the base of Neosuchia.

The development of procoelous vertebral centra and a pterygoid-bound secondary choana have been considered key transitions between early crocodyliforms and Eusuchia. However, the diversity of vertebral and palatal conditions in Mesoeucrocodylia is substantial. The shartegosuchid *Fruitachampsa callisoni* exhibits procoely throughout its vertebral column as well as a very posteriorly placed choana (although not one bound by the pterygoids). *Theriosuchus pusillus* has a relatively plesiomorphic “mesosuchian” grade palate, but exhibits procoely in at least one cervical vertebra, whereas known dorsal vertebrae are amphicoelous (Salisbury & Frey, 2001). By contrast, *Theriosuchus guimarotae* exhibits only amphicoelous centra (Schwarz & Salisbury, 2005). *Brillanceausuchus babouriensis* exhibits procoelous cervical and trunk vertebrae (Michard et al., 1990). Both *Pietraroiasuchus ormezzanoi* (see Buscalioni et al., 2011) and *Pachycheilosuchus trinquei* (see Rogers, 2003) exhibit procoelous cervical, trunk, and caudal vertebrae. In the latter taxon, the cotyles exhibit a central “dimple,” which expands into a deep concavity in the posterior caudal region (Rogers, 2003), and in former the choana is formed in a manner similar to our interpretation of *Isisfordia* with long palatines extending onto the ventral surface of the pterygoid plate. *Shamosuchus djadochtaensis* exhibits procoely in the cervical region and at least the anteriormost dorsal vertebra, although its known caudal vertebrae are amphicoelous (Pol, Turner & Norell, 2009). A single cervical vertebra associated with the type of the “mesosuchian” *Gilchristosuchus palatinus* is procoelous and the secondary choana appears similar to *Pietraroiasuchus* and *Isisfordia*.

A number of problematic Cretaceous neosuchian taxa, previously considered to be crocodylians but more recently resolved outside of Crocodylia, also exhibit procoelous centra. *Acynodon adriaticus*, initially considered to be an alligatoroid (Delfino et al., 2008) but more recently regarded as a hylaeochampsid (Rabi & Ösi, 2010; Turner & Brochu, 2010), exhibits procoely in at least one of its cervical vertebrae (Delfino et al., 2008). Vertebrae tentatively associated with the holotype of *Allodaposuchus precedens* are also all procoelous (Buscalioni et al., 2001). Most intriguingly, a referred specimen of *Susisuchus anatoceps* from the Crato Formation of Brazil exhibits procoelous cervical vertebrae but amphicoelous centra in the trunk region (Figueiredo et al., 2011). Should that attribution be correct, it suggests that *Susisuchus* exhibited a mosaic of centrum morphologies rather than a purely amphicoelous condition.

The diversity of vertebral morphologies and secondary choana formation in early neosuchian taxa suggests that a more rigorous test of the intermediate position of *Isisfordia* between other neosuchians and eusuchians requires a wide sample of neosuchian diversity. Increasing sampling among advanced neosuchians will also provide better context for a test of the hypothesis that procoely and tetraserial dorsal osteoderm shields co-evolved (Salisbury & Frey, 2001). Indeed, *Acynodon adriaticus*, *Pietraroiasuchus ormezzanoi*,  and likely *Shamosuchus djadochtaensis* exhibit tetraserial osteoderm shields. Further increasing sampling among advanced neosuchians will clarify our understanding of the sequence of palatal changes that occurred on the line to crown crocodylians, and will be critical to accurately assessing the sister groups to Crocodylia and the ancestral crocodylian morphology.

## Supplemental Information

10.7717/peerj.759/supp-1 Appendix S1 Fossil taxa used in the phylogenetic analysis

10.7717/peerj.759/supp-2 Appendix S2 Character list

## Institutional Abbreviations

ACAP-FX1: Association Culturelle, Archéologique et Paléontologique de l’Ouest Biterrois, Cruzy, Hérault, France
AMNH FARB: American Museum of Natural History, Collection of Fossil Reptiles, Amphibians, and Birds, New York, USA
CMNH: Cleveland Museum of Natural History, Cleveland, USA
CNRST-SUNY: Centre National de la Recherche Scientifique et Technologique du Mali—Stony Brook University, Stony Brook, NY
FGGUB: Faculty of Geology and Geophysics, University of Bucharest, Bucharest, Romania
FMNH: The Field Museum, Chicago, USA
IGM: Mongolian Institute of Geology, Ulaan Bataar, Mongolia
IGV: Geological Institute, Vertebrate Fossil Collections, Chinese Academy of Geological Sciences, Beijing, China
LPRP-USP: Laboratório de Paleontologia, FFCLRP, Universidade de São Paulo, Ribeirão Preto, Brazil
MCSNT: Museo Civico di Storia Naturale di Trieste, Spain
MCZ: Museum of Comparative Zoology, Harvard University, Cambridge, USA
MNHN SAM: Muséum National d’Histoire Naturelle, Gara Samani Collection, Paris, France
MPSC: Museu de Paleontologia de Santana do Cariri, Brazil
MPZ: Museo Paleontológico de la Universidad de Zaragoza, Spain
NHMUK: Natural History Museum, London, UK
PIN: Paleontological Institute Moscow, Russia
SMNK: Staatliches Museum für Naturkunde Karlsruhe, Karlsruhe, Germany
SMU: Shuler Museum of Paleontology, Southern Methodist University, Dallas, USA
QM: Queensland Museum, Brisbane, Australia
USNM: United States National Museum, Washington DC, USA

## Additional Information and Declarations

### Data Deposition

The following information was supplied regarding the deposition of related data: MorphoBank: www.morphobank.org/permalink/?P1200.
