# Supplementary material for: The monophyly of Susisuchidae (Crocodyliformes) and its phylogenetic placement in Neosuchia
Source: PeerJ. 2015 Feb 12;3:e759. doi: 10.7717/peerj.759 (PMC4330912; doi:10.7717/peerj.759)
Supplement: Appendix S1 [file peerj-03-759-s001.doc]

**The monophyly of Susisuchidae (Crocodyliformes) and its phylogenetic placement in Neosuchia**

Alan H. Turner^1^, Adam C. Pritchard^1^

^1^Department of Anatomical Sciences, Stony Brook University, Stony Brook, New York 11794, U.S.A.

**SUPPLEMENTARY APPENDIX 1.** Fossil taxa used in phylogenetic analysis. Bibliographic references and institutional accession numbers of the specimens that were scored based on firsthand examination are added after the taxon. To reproduce the taxon sampling scheme from the phylogenetic analysis, the following taxa have to be excluded from the MorphoBank dataset prior to running: *Candidodon* *itapecuruense*, *Trematochampsa*, *Stolokrosuchus* *lapparenti*, *Shamosuchus* *gradilifrons* holotype,

*Shamosuchus* *tersus*, *Shamosuchus* *ancestralis*, *Shamosuchus* *ulanicus*, and *Shamosuchus* *ulgicus*. Alternatively, you can download the Isisfordia Primary Analysis TNT file (isisfordia.matrix.final.tnt) from the “Isisfordia” folder in the “Documents” section of the MorphoBank project (www.morphobank.org/permalink/?P1200). Dataset files and the results in the form of tree files are also available with the Isisfordia folder.

*Acynodon adriaticus* – Delfino et al., 2008; MCSNT 57248

*Acynodon iberoccitanus* – Buscalioni et al., 1997; Martin, 2007; ACAP-FX1; ACAP-FX2

*Adamantinasuchus navae* – Nobre and Carvalho, 2006

*Alligator* *mississippiensis* – Clark, 1994; Brochu, 1999; FMNH 8201

*Alligatorium* – Wellnhofer, 1971; Clark, 1986, 1994

*Allodaposuchus precedens* – Buscalioni et al., 2001; Delfino et al., 2008

*Allodaposuchus cf. precedens* – Martin, 2010; MDE/CM-616

*Allodaposuchus subjuniperus* – Puértolas-Pascual et al., 2013

*Amphicotylus lucasii*– Mook, 1964; AMNH 5782

*Anatosuchus minor* – Sereno et al., 2003; Sereno and Larsson, 2009; MNN GAD17; MNN GDF603

*Araripesuchus* *buitreraensis* – Pol and Apesteguía, 2005; MPCA-PV 235

*Araripesuchus* *gomesii* – Price, 1959; AMNH 24450

*Araripesuchus* *patagonicus* – Ortega et al., 2000; MUCPv nos. 267, 269, 270, 283

*Araripesuchus tsangatsangana* – Turner, 2006; FMNH nos. PR 2297–PR 2299; UA 8720

*Araripesuchus* *wegeneri* – Buffetaut, 1981; MNHN-GDF 700; MNN GAD19

*Argochampsa krebsi* – Hua and Jouve, 2004

*Armadillosuchus arrudai* – Marinho and Carvalho, 2009

“*Asiatosuchus*” *germanicus* – Brochu, 1999, 2003; SMF Me 1801; SMNK uncat.

*Batrachomimus pastosbonensis* – Montefeltro et al., 2013

*Baurusuchus* *pachecoi* – Price, 1945; DGM 299-R

*Bernissartia* *fagesii* – Buscalioni and Sanz, 1990; Norell and Clark, 1990; Brochu, 1999

*Borealosuchus* *formidabilis* – Erickson, 1976; Brochu, 1997b, 1999

*Bretesuchus* *bonapartei* – Gasparini et al., 1993; PVL 4735

*Calsoyasuchus valliceps* – Tykoski et al., 2002; TMM uncatalogued

*Candidodon* *itapecuruense* – Carvalho, 1994

*Chimaerasuchus* *paradoxus* – Wu and Sues, 1996; IVPP V8274

*Comahuesuchus* *brachybuccalis* – Bonaparte, 1991; MACN-PV N30, MACN-PV N31; MOZ P6131; MUCPv 202

*Crocodylus* *niloticus* – Clark, 1994; Brochu, 1999; FMNH 17157, FMNH 217153

*Dakosaurus andiniensis* – Vignaud and Gasparini, 1996; MHNSR PV 344; MOZ 6146P

*Dakosaurus maximus* – Fraas, 1902; NHMUK R486; BSP AS.VI.1; SMNS 8203

*Dibothrosuchus* *elaphros* – Wu and Chatterjee, 1993; IVPP V 7907

*Diplocynodon hantoniensis* – Brochu, 1999; NHMUK nos. 25178, 25199, 25245, 30206, 30210, 30219, 30236, 30250, 30289, 30362, 30368, 30391–30394, 30397, 30402, 30414, R1046, R1050, R5230

*Dyrosaurus* – Buffetaut, 1978; Clark, 1986, 1994; Jouve, 2005; BSP 1993.IX.400

*Edentosuchus tienshanensis* – Young, 1973; GMPKU-P 200101; IVPP V 3236

*Elosuchus cherifiensis* – Adams, 2013

*Eothoracosaurus mississippiensis* – Brochu, 2004; MSU 3293; PPM p2000.1.60

*Eutretauranosuchus* *delfsi* – Mook, 1967; Clark, 1986, 1994; AMNH 570; CMNH 8028

*Fruitachampsa* – Clark, 1985, 1994; LACM 120455a

*Gavialis* *gangeticus* – Clark, 1994; Brochu, 1999; FMNH 82681, FMNH 98864; MLP s/n

*Geosaurus araucanensis* – Gasparini and Dellapé, 1976; MACN-PV N 64, MACN-PV N 95; MLP nos. 72-IV-7-1–72-IV-7-4, 86-XI-5-7

*Geosaurus suevicus* – Fraas, 1902; SMNS [Fraas specimen]

Glen Rose Form – Brochu 1997a, 1999; MCZ 4453; USNM 22039,

*Gobiosuchus* *kielanae* – Osmólska, 1972; Osmólska et al., 1997; ZPAL nos. MgR-II/67–MgR-II/71

*Goniopholis simus* – Mook, 1942; Clark, 1986, 1994; Salisbury et al., 1999; NHMUK 841098

*Goniopholis baryglyphaeus* – Schwarz, 2002

*Gracilisuchus* *stipanicicorum* – Romer, 1972; MCZ 4118; PVL 4597, PVL 4612

*Hamadasuchus rebouli* – Larsson and Sues, 2007; ROM 52620

*Hemiprotosuchus* *leali* – Bonaparte, 1971; PVL 3829

*Hsisosuchus* *chungkingensis* – Young and Chow, 1953; Li et al., 1994; Wu et al., 1994b; cast of CNM V 1090

*Hylaeochampsa* *vectiana* – Clark and Norell, 1992; BMNH R177

*Hyposaurus rogersii* – Troxell, 1925; Denton et al., 1997; YPM 764, YPM 985

*Iberosuchus* *macrodon* – Antunes, 1975; Ortega et al., 2000; STUS specimens

*Iharkutosuchus makadii* – Osi, 2008; MTM nos. 2006.52.1, 2006.53.1, 2006.54.1, 2006.55.1, 2006.56.1, 2006.57.1, 2006.65.1, 2006.66.1, 2006.58.1

*Isisfordia duncani* – Salisbury et al., 2006; QM nos. F36211, F44320, F44319, F34642

*Kaprosuchus saharicus* – Sereno and Larsson, 2009; MNN IGU12

Kayenta Form – Clark, 1986; UCMP nos. 97638, 125359, 125871

*Leidyosuchus canadensis* – Brochu, 1997b, 1999, 2003; Wu et al., 2001b; AMNH 5352; NMC 2279; TMP 74.10.8

*Libycosuchus brevirostris* – Stromer, 1914; BSP 1912.VIII.574

*Lomasuchus palpebrosus* – Gasparini et al., 1991; MOZ 4084 PV

*Mahajangasuchus insignis* – Buckley and Brochu, 1999; Turner and Buckley, 2008; FMNH nos. PR 2389, PR 2448–PR 2450; UA nos. 8654, 9046, 9047, 9737

*Malawisuchus* *mwakasyungutiensis* – Clark et al., 1989; Gomani, 1997; MAL 45, MAL 49

*Mariliasuchus* *amarali* – Carvalho and Bertini, 1999; MNRJ 6298-V, MNRJ 6756-V; MZSP-PV 50, MZSP-PV 51

*Meridiosaurus vallisparadisi*– Adams, 2013

*Metriorhynchus casamiquelai* – Gasparini and Diaz, 1977; cast of MGHF 1.08573

*Metriorhynchus superciliosus* – Blainville, 1853; Wenz, 1968; AMNH 997, SMNS 10116

*Montealtosuchus arrudacamposi* – Carvalho et al., 2007

*Notosuchus* *terrestris* – Gasparini, 1971; MACN-PV RN nos. 1037, 1040–1044; MLP nos. 64-IV-16-1, 64-IV-16-5, 64-IV-16-6, 64-IV-16-10–64-IV-16-13, 64-IV-16-23; MPCA-PV 249, MPCA-PV250; MUCPv 287

*Oceanosuchus boecensis* – Adams, 2013

*Orthosuchus* *stormbergi* – Nash, 1975; SAM-K 409

*Pachycheilosuchus trinquei* – Rogers, 2003

*Paluxysuchus newmani* – Adams, 2013

*Paralligator gradilifrons* – Konzhukova, 1954; PIN 554-1; PIN 551-29/1; PIN 551-29/3; PIN 551-29/7; PIN 551-29/20; PIN 551-29/27; PIN 3458/501; PIN 3140-502; PIN 3141-501;

*Paralligator major* – Efimov, 1981; PIN 3726/501

*Peirosaurus* *torminni* – Price, 1955; Gasparini et al., 1991; MOZ 1750 PV

*Pelagosaurus* *typus* – Eudes-Deslongchamps, 1864; NHMUK R32599; BSP 1925.I.34, BSP 1990.VIII.68; MB 1925.1, MB R.2883; SMNS 8666, SMNS 80066

*Pholidosaurus* *purbeckensis* – Owen, 1878; Clark, 1986, 1994; Salisbury, 2002; BMNH R3414

*Pristichampsus vorax* – Brochu, 1999; FMNH nos. PR 74, PR 399, PR 479; UCMP 154329

*Protosuchus* *richardsoni* – Colbert and Mook, 1951; AMNH 3024; MCZ 6727; UCMP 130860, UCMP 131827

*Rhabdognathus* – Brochu et al., 2002; CNRST-SUNY 190

*Rugosuchus nonganensis* – Wu et al., 2001a

*Sarcosuchus imperator* – Broin and Taquet, 1966; Sereno et al., 2001; MNN 603, MNN 604

*Shamosuchus djadochtaensis* – Mook, 1924; AMNH 6412; IGM 100/1195

*Shantungosuchus hangjinensis* – Wu et al., 1994a

*Sichuanosuchus* *shuhanensis* – Wu et al., 1997; IVPP V 10594

*Simosuchus* *clarki* – Buckley et al., 2000; FMNH nos. PR 2596–2599; UA nos. 8679, 9753, 9754, 9762, 9776

*Sokotosuchus ianwilsoni* – Halstead 1975; Buffetaut, 1979; Clark, 1986, 1994

*Sphagesaurus* *huenei* – Price, 1950; Pol, 2003; RCL 100

*Sphagesaurus montealtensis* – Andrade and Bertini, 2008a

*Steneosaurus bollensis* – Jaeger, 1828; Westphal, 1962; AMNH 5138; BSP nos. 1890.I.510, 1945.XV.1, 1949.XV.1, 1972.V.11, 1973.VII.592; GPIT Re.1193-6, GPIT Re.1193-12; MB nos. 1878.262, 1921.12, R.1953; SMNS nos. 115, 4554, 9427, 9428, 15951, 16848, 17484, 18878, 20280, 20282, 20283, 53422

*Stolokrosuchus lapparenti* – Larsson and Gado, 2000; MNN GDF600

*Sunosuchus junggarensis* – Wu et al., 1996

*Susisuchus anatoceps* – Salisbury et al., 2003; SMNK 3804 PAL

*Terminonaris robusta* – Mook, 1934; Wu et al., 2001c; AMNH 5849, AMNH 5850

*Terrestrisuchus* *gracilis* – Crush, 1984

*Theriosuchus* *pusillus* – Owen, 1879; Clark, 1986, 1994; Ortega et al., 2000; NHMUK R48328, NHMUK R48330

*Theriosuchus guimarotae* – Schwarz and Salisbury, 2005

*Theriosuchus sympiestodon* – Martin et al., 2010, 2014

*Uberabasuchus terrificus* – Carvalho et al., 2004

*Uruguaysuchus* *aznarezi* – Rusconi, 1933

*Wannchampsus kirpachi* – Adams, 2014

*Yacarerani boliviensis* – Novas et al., 2009

*Zaraasuchus shepardi* – Pol and Norell, 2004b; IGM 100/1321

*Zosuchus* *davidsoni* – Pol and Norell, 2004a; IGM nos. 100/1304–100/1308

**Institutional Abbreviations**—**ACAP-FX1**, Association Culturelle, Archéologique et Paléontologique de l’Ouest Biterrois, Cruzy, Hérault, France; **AMNH**, American Museum of Natural History, New York, USA; DGM, Departamento de Produção Mineral, Rio de Janeiro, Brazil; **BPI**, Bernard Price Institute, Johannesburg, South Africa; **BSP**, Bayerische Staatssammlung für Paläontologie und Geologie, Münich, Germany; **CMNH**, Cleveland Museum of Natural History, Cleveland, USA; **CNM**, Chongqing Natural Museum, Sichuan, People‘s Republic of China; **CNRST-SUNY**, Centre National de la Recherche Scientifique et Technologique, Mali - Stony Brook University, New York, USA; **DGM**, Departamento de Produção Mineral, Rio de Janeiro, Brazil; **FC-DPV**, Colección de Vertebrados Fósiles, Facultad de Ciencias, Montevideo, Uruguay; **FFCLRP**, Universidade de São Paulo, Ribeirão Preto, Brazil; **FGGUB**, Faculty of Geology and Geophysics, University of Bucharest, Bucharest, Romania; **FMNH**, Field Museum of Natural History, Chicago, Illinois, USA; **GMPKU-P**, School of Earth and Space Sciences, Peking University, Beijing, People‘s Republic of China; **GPIT**, Institut und Museum für Geologie und Paläontologie, Universitat Tübingen, Tübingen, Germany; **GSP-UM**, Geological Survey of Pakistan-University of Michigan collection, Quetta, Pakistan; **HLMD**, Hessisches Landesmuseum, Darmstadt, Germany; **IGM**, Mongolian Institute of Geology, Ulaan Bataar, Mongolia; **IGV**, Geological Institute, Vertebrate Fossil Collections, Chinese Academy of Geological Sciences, Beijing, China; **IVPP**, Institute of Vertebrate Paleontology and Paleoanthropology, Beijing, China; **LACM**, Natural History Museum of Los Angeles County, Los Angeles, California, USA; **MACN**, Museo Argentino de Ciencias Naturales, Buenos Aires, Argentina; **MAL**, Malawi Department of Antiquities, Malawi; **MB**, Institut für Palaontologie, Museum fur Naturkunde, Humbolt-Universitat, Berlin, Germany; **MCF**, Museo Carmen Funes, Plaza Huincul, Argentina; **MCSNT**, Museo Civico di Storia Naturale di Trieste, Spain;**MCZ**, Museum of Comparative Zoology, Harvard University, Cambridge, Massachusetts, USA; **MDE**, Musée des Dinosaures D‘Espéraza, France; **MGHF**, Museo Geológico H. Fuenzalida, Universidad Católica del Norte, Antofagasta, Chile; **MHNSR**, Museo de Historia Natural de San Rafael, San Rafael, Argentina; **MLP**, Museo de La Plata, La Plata, Argentina; **MMP**, Museo de Historia Natural ―Galileo Scagliaǁ‖, Mar del Plata, Argentina; **MNHN**, Museum National d‘Histoire Naturelle, Paris, France; **MNK**, Museo Noel Kempff Mercado, Santa Cruz de la Sierra, Bolivia; **MNN**, Musée National du Niger; **MNRJ**, Museu Nacional, Universidade Federal do Rio de Janeiro, Rio de Janeiro, Brazil; **MOZ**, Museo Profesor J. Olsacher, Zapala, Argentina ;**MPCA-PV**, Museo Carlos Ameghino, Cipoletti, Argentina; **MPEF**, Museo Paleontológico Egidio Feruglio, Trelew, Argentina; **MPMA**, Museu de Paleontologia de Monte Alto, Brazil; **MPZ**, Museo Paleontológico de la Universidad de Zaragoza, Spain; **MSU**, Dunn-Seiler Museum, Mississippi State University, Starkville, USA; **MTM**, Hungarian Natural History Museum, Budapest, Hungary; **MUC-PV**, Museo de Geología y Paleontología, Universidad Nacional del Comahue, Neuquén, Argentina; **MZSP**, Museu Zoologia, Universidade de São Paulo, São Paulo, Brazil; **NHMUK**, Natural History Museum, London, United Kingdom; **NMC**, Canadian Museum of Nature, Ottawa, Ontario, Canada; **PPM**, Pink Palace Museum, Memphis, Tennessee, USA; **PVL**, Instituto Miguel Lillo, Tucumán, Argentina; **RCL**, Museo de Ciencias Naturales, Pontificia Universidade Catolica de Minas Gerais, Brazil; **SAM**, Iziko-South African Museum, Cape Town, South Africa; **UA**, University of Antananarivo, Madagascar; **SMF**, Senckenberg Museum, Frankfurt, Germany; **SMNK**, Staatliches Museum fur Naturkunde, Karlsruhe, Germany; **SMNS**, Staatliches Museum fur Naturkunde, Stuttgart, Germany; **SMU**, Shuler Museum of Paleontology, Southern Methodist University, Dallas, USA; **STUS**, Sala de las Tortugas, Universidad de Salamanca, Salamanca, Spain; **TMM**, Texas Memorial Museum, Austin, USA; **TMP**, Royal Tyrrell Museum of Palaeontology, Drumheller, Alberta, Canada; **QM**, Queensland Museum, Brisbane, Australia; **UAM**, Universidad Autónoma de Madrid, Spain; **UCMP**, University of California Museum of Paleontology, Berkeley, California, USA; **UFRJ**, Universidade Federal do Rio de Janeiro, Departamento de Geologia, Brazil; **URC**, Museu de Paleontologia e Estratigrafi a Prof. Dr. Paulo Milton Barbosa Landim, Universidade Estadual Paulista, Rio Claro, Brazil; **USNM**, United States National Museum, Smithsonian Institution, Washington, DC; **YPM**, Peabody Museum of Natural History, Yale University, New Haven, Connecticut, USA; **ZDM**, Zigong Dinosaur Museum, Zigong, China; **ZPAL**, Instytut Paleobiologii PAN, Warszawa, Poland.

**Supplementary Literature Cited**

Adams TL. 2013. A new neosuchian crocodyliform from the Lower Cretaceous (Late Aptian) Twin Mountains Formation of North-Central Texas. Journal of Vertebrate Paleontology 33: 85–101.

Adams TL. 2014. Small crocodyliform from the Lower Cretaceous (Late Aptian) of Central Texas and its systematic relationship to the evolution of Eusuchia. Journal of Paleontology 88: 1031–1049.

Andrade, M. B., and R. J. Bertini. 2008a. A new *Sphagesaurus* (Mesoeucrocodylia: Notosuchia) from the Upper Cretaceous of Monte Alto City (Bauru Group, Brazil), and a revision of the Sphagesauridae. Historical Biology 20:101–136.

Andrade, M.B., and Bertini, R. J. 2008b.Morphology of the dental carinae in *Mariliasuchus amarali* (Crocodylomorpha, Notosuchia) and the pattern of tooth serration among basal mesoeucrocodylia. Arquivos do Museu Nacional, Rio de Janeiro 66:63–82.

Andrade, M. B., Edmonds, R., Benton, M. J., and Schouten, R. 2011. A new Berriasian species of *Goniopholis* (Mesoeucrocodylia, Neosuchia) from England, and a review of the genus. Zoological Journal of the Linnean Society 163:S66–S108.

Antunes, M. T. 1975. *Iberosuchus*, crocodile Sebecosuchien nouveau, l'Eocene iberique au Nord de la Chaine Centrale, et l'origine du canyon de Nazare. Comunicaçoes dos Serviços Geológicos de Portugal 59:285–330.

Blaniville, H. M. D. de. 1853. Lettre sur les Crocodiliens vivants et fossils. Memoires de la Société Linneenne de Normandie, Caen 9:109–120.

Bonaparte, J. F. 1971. Los tetrápodos del sector superior de la formación Los Colorados, La Rioja, Argentina. Opera Lilloana 22:1–183.

Bonaparte, J. F. 1991. Los vertebrados fósiles de la formación Río Colorado, de la ciudad de Neuquén y sus cercanías, Cretácico superior, Argentina. Revista del Museo Argentino de Ciencias Naturales ―Bernardino Rivadaviaǁ‖ Paleontología 4:17–123.

Bremer, K. 1994. Branch support and tree stability. Cladistics 10:295–304. Brochu, C. A. 1997a.Fossils, morphology, divergence timing, and the phylogenetic relationships of *Gavialis*.Systematic Biology 46:479–522.

Brochu, C. A. 1997b.A review of "*Leidyosuchus*" (Crocodyliformes, Eusuchia) from the Cretaceous through Eocene of North America.Journal of Vertebrate Paleontology 17:679– 697.

Brochu, C. A. 1999. Phylogenetics, taxonomy, and historical biogeography of Alligatoroidea.Memoir of the Society of Vertebrate Paleontology 4:1–92.

Brochu, C. A. 2003. Phylogenetic approaches toward Crocodylian history. Annual Review of Earth and Planetary Sciences 31:357–397.

Brochu, C. A. 2004.A new Late Cretaceous gavialoid crocodylian from eastern North America and the phylogenetic relationships of thoracosaurs.Journal of Vertebrate Paleontology 24:610–633.

Brochu, C. A., M. L. Bouaré, F. Sissoko, E. M. Roberts, and M. A. O‘Leary. 2002. A dyrosaurid crocodyliform braincase from Mali. Journal of Paleontology 76:1060–1071.

Broin, F. de, and P. Taquet. 1966. Découverte d‘un crocodilien nouveau dans le Crétace inférieur du Sahara. Comptes Rendus de l‘Académie de Sciences, Series D 262:2326– 2329.

Buckley, G. A. and C.A. Brochu. 1999. An enigmatic new crocodile from the Upper Cretaceous of Madagascar; pp. 149–175 in D. M. Unwin (ed.), Cretaceous Fossil Vertebrates.Special Papers in Palaeontology, 60.

Buckley, G. A., C. A. Brochu, D. W. Krause, and D. Pol. 2000.A pug-nosed crocodyliform from the Late Cretaceous of Madagascar.Nature 405:941–944.

Buffetaut, E. 1978. Les Dyrosauridae (Crocodylia, Mesosuchia) des phosphates de l‘Eocène inferieur de Tunisie: *Dyrosaurus*, *Rhabdognathus*, *Phosphatosaurus*.

Geologie Mediterranéenne 5:237–256.

Buffetaut, E. 1979.*Sokotosuchus ianwilsoni* and the evolution of the dyrosaurid crocodilians.Nigerian Field Monographs 1:31–41.

Buffetaut, E. 1981. Die biogeographische Geschichte der Krokodilier, mit Beschreibung einer neuen Art, *Araripesuchus wegeneri*. Geologischen Rundschau 70:611–624.

Buffetaut, E. 1994.A new crocodilian from the Cretaceous of southern Morocco.Comptes Rendus de l‘Académie Des Sciiences 319:1563–1568.

Buffetaut E, and C. Marshall. 1991. A new crocodilian, *Sebecus querejazus*, nov. sp. (Mesosuchia, Sebecidae) from the Santa Lucía Formation (Early Paleocene) at Vila Vila, Southcentral Bolivia; pp. 545–557 in R. Suárez-Soruco (ed.), Fósiles y Facies de Bolivia, Volume I Vertebrados. Revista Técnica de YPFB. Santa Cruz.

Busbey, A. B. III. 1986. New material of *Sebecus* cf. *huilensis* (Crocodilia: Sebecosuchidae) from the Miocene La Venta Formation of Colombia. Journal of Vertebrate Paleontology 6:20–27.

Busbey, A. B. III, and Gow, C. E. 1984. A new protosuchian crocodile from the Upper Triassic Elliot Formation of South Africa. Palaeontologia Africana 25:127-149

Buscalioni, A. D., and J. L. Sanz. 1988. Phylogenetic relationships of the Atoposauridae (Archosauria, Crocodylomorpha). Historical Biology 1:233–250.

Buscalioni, A. D., and J. L. Sanz. 1990. The small crocodile *Bernissartia fagesii* from the Lower Cretaceous of Galve (Teruel, Spain). Bulletin de l'Institut Royal des Sciences Naturelles de Belgique, Sciences de la Terre 60:129–150.

Buscalioni AD, Ortega F, Vasse D. 1997. New crocodiles (Eusuchia: Alligatoroidea) from the Upper Cretaceous of southern France. *Comptes Rendus de Academie des Sciences de Paris, Sciences de la terre et des planetes* 325:525–530.

Buscalioni AD, Ortega F, Weishampel DB, Jianu CM. 2001. A revision of the crocodyliform *Allodaposuchus precedens* from the Upper Cretaceous of the Hateg Basin, Romania. Its relevance to the phylogeny of Eusuchia. *Journal of Vertebrate Paleontology* 21:74–86.

Campos, D. A., J. M. Suarez, D. Riff, and A. W. A. Kellner. 2001. Short note on a new Baurusuchidae (Crocodyliformes, Metasuchia) from the Upper Cretaceous of Brazil. Boletim do Museu Nacional, Nova Série, Geologia 57:1–7.

Carvalho, I. S. 1994. *Candidodon*: um crocodilo com heterodontia (Notosuchia, Cretáceo Inferior - Brazil). Anais da Academia Brasileira de Ciencias 66:331–346.

Carvalho, I. S., and R. J. Bertini. 1999. *Mariliasuchus*, um novo Crocodylomorpha (Notosuchia) do Cretáceo da bacia Bauru, Brasil. Revista Geologia Colombiana 24:83– 105.

Carvalho, I. S., A. C. A. Campos, and P. H. Nobre. 2005. *Baurusuchus salgadoensis*, a new Crocodylomorpha from the Bauru Basin (Cretaceous), Brazil. Gondwana Research 8:11–30.

Carvalho, I. S., L. C. B. Ribeiro, and L. S. Avilla. 2004. *Uberabasuchus terrificus* sp. nov., a new Crocodylomorpha from the Bauru Basin (Upper Cretaceous), Brazil. Gondwana Research 7:975–1002.

Carvalho, I. S., Vasconcellos, F. M., and Tavares, S. A. S. 2007. *Montealtosuchus arrudacamposi*, a new peirosaurid crocodile (Mesoeucrocodylia) from the Late Cretaceous Adamantina Formation of Brazil. Zootaxa1607:35–46.

Carvalho, I. S., Teixeira, V. P. A., Ferraz, M. L. F., Ribeiro, L. C. B., Martinelli, A. G., et al. 2011. *Campinasuchus dinizi* gen. et sp. nov., a new Late Cretaceous baurusuchid (Crocodyliformes) from the Bauru Basin, Brazil. Zootaxa 2871:19-42.

Clark, J. M. 1985. A new crocodylomorph from the Late Jurassic Morrison Formation of western Colorado, with a discussion of relationships within the 'Mesosuchia.' M.S. thesis, University of California, Berkeley, 106 pp.

Clark, J. M. 1986.Phylogenetic relationships of the crocodylomorph archosaurs.Ph.D.dissertation, University of Chicago, 556 pp.

Clark, J. M. 1994. Patterns of evolution in Mesozoic Crocodyliformes; pp. 84–97 in N. C. Fraser and H.-D. Sues (eds.), In the shadow of dinosaurs. Cambridge University Press, Cambridge.

Clark, J. M., and M. A. Norell. 1992. The early Cretaceous crocodylomorph *Hylaeochampsa vectiana* from the Wealden of the Isle of Wight. American Museum Novitates 3032:1–19.

Clark, J. M., L. L. Jacobs, and W. R. Downs. 1989. Mammal–like dentition in a Mesozoic crocodylian. Science 244:1064–1066.

Coddington, J. A., and N. Scharff. 1994. Problems with zero-length branches. Cladistics 10:415–423.

Colbert, E. C. 1946.*Sebecus*, representative of a peculiar suborder of fossil Crocodilia from Patagonia.Bulletin of the American Museum of Natural History 87:221–270.

Colbert, E. C., and C. C. Mook. 1951. The ancestral crocodile *Protosuchus*. Bulletin of the American Museum of Natural History 97:143–182.

Crush, P. J. 1984. A late Upper Triassic sphenosuchid crocodilian from Wales.Palaeontology 27:131–157.

Delfino M, Codrea V, Folie A, Dica P, Godefroit P, Smith T. 2008. A complete skull of *Allodaposuchus precedens* Nopcsa, 1928 (Eusuchia) and a reassessment of the morphology of the taxon based on the Romanian remains. *Journal of Vertebrate Paleontology* 28:111–122.

Denton, R. K., J. L. Dobie, and D. C. Parris. 1997. The marine crocodilian *Hyposaurus* in North America; pp. 375–396 in J. M. Callaway and E. L. Nicholls (eds.), Ancient Marine Reptiles. Academic Press, San Diego.

Efimov MB. 1981. New paralligatorids from the Upper Cretaceous of Mongolia. Trudy Sovmestnoi Sovetsko-Mongol'skoi Paleontologicheskoi Ekspeditsii 15:26–28.

Erickson, B. R. 1976. Osteology of the early eusuchian crocodile *Leidyosuchus formidabilis*, sp. nov. Monograph of the Science Museum of Minnesota Paleontology 2:1– 61.

Eudes-Deslongchamps, E. 1863. Memoires sur les téléosauriens de l'epoque Jurassique du département du Calvados. Memoires de la Société Linneenne de Normandie 13:1–138.

Farlow, J. O., G. R. Hurlburt, R. M. Elsey, A. R. C. Britton, and W. J. Langston. 2005. Femoral dimensions and body size of *Alligator mississippiensis*: estimating the size of extinct mesoeucrocodylians. Journal of Vertebrate Paleontology 25:354–369.

Farris, J. S., V. A. Albert, M. Källersjö, D. Lipscomb, and A. C. Kludge. 1996. Parsimony jackknifing outperforms neighbor-joining. Cladistics 12:99–124.

Felsenstein, J. 1985. Confidence limits on phylogenies: an approach using the bootstrap. Evolution 39:783–791.

Fraas, E. 1902. Die Meer Crocodilier (Thalattosuchia) des oberen Jura unter spezieller Berücksichtigung von *Dacosaurus* und *Geosaurus*. Palaeontographica 49:1–72.

Frey, E. 1988. Das Tragsystem der Krocodile - eine biomechanische und phylogenetische Analyse. Stuttgarter Beitrage zur Naturkunde (serie A) 426:1–60.

Gasparini, Z. 1971. Los Notosuchia del Cretácico de América del Sur como un nuevo infraorden de los Mesosuchia (Crocodilia). Ameghiniana 8:83–103

Gasparini, Z. 1984. New Tertiary Sececosuchia (Crocodylia: Mesosuchia) from Argentina.Journal of Vertebrate Paleontology 4:85–95.

Gasparini, Z., and D. Dellapé. 1976. Un nuevo cocodrilo marino (Thalattosuchia, Metriorhynchidae) de la Formación Vaca Muerta (Jurásico, Tithoniano) de la Provincia de Neuquén. Actas I Congreso Geológico Chileno, Santiago 1976:C1–C21.

Gasparini, Z., and G. Díaz. 1977. *Metriorhynchus casamiquelai* n. sp. (Crocodilia, Thalattosuchia) a marine crocodile from the Jurassic (Callovian) of Chile,

South America. Neues Jahrbuch fur Geologie und Palaontologie, Abhandlungen 153:341– 360.

Gasparini, Z., L. Chiappe, and M. Fernández. 1991. A new Senonian peirosaurid (Crocodylomorpha) from Argentina and a synopsis of the South American Cretaceous crocodilians. Journal of Vertebrate Paleontology 11:316–333.

Gasparini, Z., M. Fernández, and J. Powell. 1993. New Tertiary sebecosuchians (Crocodylomorpha) from South America: phylogenetic implications. Historical Biology 7:1– 19.

Goloboff, P. A., J. S. Farris, and K. C. Nixon. 2008b. TNT, a free program for phylogenetic analysis.Cladistics 24:774–786.

Goloboff, P. A., J. M. Carpenter, J. S. Arias, and D. R. Miranda-Esquivel. 2008a. Weighting against homoplasy improves phylogenetic analysis of morphological data sets. Cladistics 24:1–16.

Goloboff, P. A., J. S. Farris, M. Källersjö, B. Oxelman, M. J. Ramirez, and C. A. Szumik. 2003. Improvements to resampling measures of group support. Cladistics 19:324–332.

Gomani, E. M. 1997.A crocodyliform from the Early Cretaceous Dinosaur Beds, Northern Malawi.Journal of Vertebrate Paleontology 17:280–294.

Halstead, L. B. 1975.*Sokotosuchus ianwilsoni* n.g., n.sp., a new teleosaur crocodile from the Upper Cretaceous of Nigeria. Nigerian Journal of Mineral Geology 11:101–103.

Hua, S., and S. Jouve. 2004. A primitive marine gavialoid from the Paleocene of Morocco. Journal of Vertebrate Paleontology 24:341–350.

Iori, F. V., and Carvalho, I. S. 2009. *Morrinhosuchus luziae*, um novo Crocodylomorpha Notosuchia da Bacia Bauru, Brasil. Revista Brasileira de Geociências 39:717-725.

Iori, F. V., and Carvalho, I. S. 2011. *Caipirasuchus paulistanus*, a new sphagesaurid (Crocodylomorpha, Mesoeucrocodylia) from the Adamantina Formation (Upper Cretaceous, Turonian–Santonian), Bauru Basin, Brazil. Journal of Vertebrate Paleontology 31: 1255-1264.

Jaeger, G. F. 1828. Über die fossile Reptilien, welche in Württemberg aufgefunden wordensind. Metzler, Stuttgart, 48 pp.

Jouve, S. 2005. A new description of the skull of *Dyrosaurus phosphaticus* (Thomas, 1893) (Mesoeucrocodylia: Dyrosauridae) from the lower Eocene of North Africa.Canadian Journal of Earth Sciences 42:323–337.

Kellner, A. W. A., Pinheiro, A. E. P., Azevedo, S. A. K., Henriques, D. D. R., Carvalho, L. B., et al. 2009. A new crocodyliform from the Alcântara Formation (Cenomanian), Cajual Island, Brazil. Zootaxa 2030:49-58.

Kellner, A. W. A., Campos, D. A., Riff, D., and Andrade, M. B. 2011a. A new crocodylomorph (Sphagesauridae, Notosuchia) with horn-like tubercles from Brazil. Zoological Journal of the Linnean Society 163:S57-S65.

Kellner, A. W. A., Figueiredo, R. G., Azevedo, S. A. K., and Campos, D. A. 2011b. A new cretaceous notosuchian (Mesoeucrocodylia) with bizarre dentition from Brazil. Zoological Journal of the Linnean Society 163:S109-S115.

Konzhukova ED. 1954. [New fossil crocodilian from Mongolia]. Trudy Paleontologicheskogo Instituta ANSSSR 48: 171–194.

Krause, D. W., and N. J. Kley. 2010. *Simosuchus clarki* (Crocodyliformes: Notosuchia) from the Late Cretaceous of Madagascar. Memoirs of the Society of Vertebrate Paleontology 10:1–236.

Kuhn, O. 1968. Die Vorzeitlichen Krokodile. Verlag Oeben, München, 124 pp + 68 Abb.

Langston, W. 1965.Fossil crocodilians from Colombia and the Cenozoic history of the Crocodilia in South America.University California Publication Geological Sciences 52:1– 157.

Langston, W., and Z. Gasparini. 1997. Crocodilians, *Gryposuchus*, and the South American gavials; pp. 113–154 in R. Kay, R. Madden, R. Cifelli, and J. Flynn (eds.), Vertebrate Paleontology in the Neotropics. The Miocene Fauna of La Venta, Colombia. Smithsonian Institution Press, Washington, D.C..

Larsson, H. C. E., and B. Gado. 2000. A new Early Cretaceous crocodyliform from Niger. Neues Jahrbuch für Geologie und Paläontologie, Abhandlungen 217:131–141.

Larsson, H. C. E., and H.-D. Sues. 2007. Cranial osteology and phylogenetic relationships of *Hamadasuchus rebouli* (Crocodyliformes: Mesoeucrocodylia) from the Cretaceous of Morocco. Zoological Journal of the Linnean Society 149:533–567.

Li, J., X.-C. Wu, and X. Li. 1994. New material of *Hsisosuchus chungkingensis* from Sichuan, China.Vertebrata PalAsiatica 32:107–126.

Marinho, T. S., and Carvalho, I. S. 2009. An armadillo-like sphagesaurid crocodyliform from the Late Cretaceous of Brazil. Journal of South American Earth Sciences 27:36-41.

Martin JE. 2007. New material of the Late Cretaceous globidontan *Acynodon iberoccitanus* (Crocodylia) from southern France. *Journal of Vertebrate Paleontology* 27:362–372.

Martin JE. 2010. *Allodaposuchus* Nopsca, 1928 (Crocodylia, Eusuchia), from the Late Cretaceous of Southern France and its relationships to Alligatoroidea. *Journal of Vertebrate Paleontology* 30:756–767.

Martin JE, Rabi M, Csiki Z. 2010. Survival of *Theriosuchus* (Mesoeucrocodylia: Atoposauridae) in a Late Cretaceous archipelago: a new species from the Maastrichtian of Romania. Naturwissenschaften 97:845–854.

Martin JE, Rabi M, Csiki Z, Vasile S. 2014. Cranial morphology of *Theriosuchus sympiestodon* (Mesoeucrocodylia, Atoposauridae) and the widespread occurrence of *Theriosuchus* in the Late Cretaceous of Europe. Journal of Paleontology 88: 444–456.

Martinelli, A. G. 2003.New cranial remains of the bizarre notosuchid *Comahuesuchus brachybuccalis* (Archosauria, Crocodyliformes) from the Late Cretaceous of Río Negro Province (Argentina).Ameghiniana 40:559–572.

Martinelli, A. G., Sertich, J. J. W., Garrido, A. C., Praderio, A. M. 2012. A new peirosaurid from the Upper Cretaceous of Argentina: Implications for specimens referred to *Peirosaurus torminni* Price (Crocodyliformes: Peirosauridae). Cretaceous Research 37:191-200.

Meers, M.B. 2003. Crocodylian forelimb musculature and its relevance to Archosauria. The Anatomical Record Part A 274A:891–916.

Mook, C.C. 1924. A new crocodilian from Mongolia.American Museum Novitates 117:1–5.

Mook, C.C. 1934.A new species of *Teleorhinus* from the Benton Shales.American Museum Novitates 702:1–11.

Mook, C.C. 1942. Skull characters of *Amphicotylus lucasii* Cope.American Museum Novitates 1202:1–5.

Mook, C.C. 1964. New species of *Goniopholis* from the Morrison of Oklahoma.Oklahoma Geology Notes 24:283–287.

Mook, C.C. 1967.Preliminary description of a new goniopholid crocodilian.Kirtlandia 2:1– 10.

Montefeltro, F. C., Larsson, H. C. E., and Langer, M. C. 2011. A new baurusuchid (Crocodyliformes, Mesoeucrocodylia) from the Late Cretaceous of Brazil and the phylogeny of Baurusuchidae. PLoS ONE 6: 1-26.

Nascimento, P. M., and H. Zaher. 2010. A new species of *Baurusuchus* (Crocodyliformes, Mesoeucrocodylia) from the Upper Cretaceous of Brazil, with the first complete postcranial skeleton described for the family Baurusuchidae. Papéis Avulsos de Zoologia 50:323–361.

Nascimento, P.M., and Zaher, H. 2011. The skull of the Upper Cretaceous baurusuchid crocodile *Baurusuchus albertoi* (Crocodyliformes, Mesoeucrocodylia), and its phylogenetic affinities. Zoological Journal of the Linnean Society 163:S116-S131.

Nash, D. S. 1975. The morphology and relationships of a crocodilian, *Orthosuchus stormbergi*, from the Upper Triassic of Lesotho.Annals of the South African Museum 67:227–329.

Nobre, P. H., and I. S. Carvalho.2001.Morfologia do crânio de *Candidodon itapecuruense*, um Crocodylomorpha (Notosuchia) cretácico do Brasil. Revista Brasileira de Paleontologia 2:144–145.

Nobre, P. H., and Carvalho, I. S. 2006. *Adamantinasuchus navae*: a new Gondwanan Crocodylomorpha (Mesoeucrocodylia) from the Late Cretaceous of Brazil. Gondwana Research 10:370-378.

Norell, M. A. 1988. Cladistic approaches to paleobiology as applied to the phylogeny of alligatorids. Ph.D. dissertation, Yale University, New Haven, Connecticut, 544 pp.

Norell, M. A., and J. M. Clark. 1990. A reanalysis of *Bernissartia fagesii*, with comments on its phylogenetic position and its bearing on the origin and diagnosis of the Eusuchia. Bulletin de L'Institut Royal des Sciences Naturalles de Belgique, Sciences de la Terre 60:115–128.

Novas, F. E., Pais, D. F., Pol, D., Carvalho, I. S., Scanferla, A., et al. 2009. Bizarre notosuchian crocodyliform with associated eggs from the Upper Cretaceous of Bolivia. Journal of Vertebrate Paleontology 29:1316-1320.

O’Connor, P. M., Sertich, J. J. W., Stevens, N. J., Roberts. E. M., Gottfried, M. D., et al. 2010. The evolution of mammal-like crocodyliforms in the Cretaceous Period of Gondwana. Nature 466: 748- 751.

Ortega, F. 2004. Historia evolutiva de los cocodrilos Mesoeucrocodylia. PhD dissertation, Universidad Autónoma de Madrid, Madrid, 350 pp.

Ortega, F., A. D. Buscalioni, and Z. Gasparini. 1996. Reinterpretation and new denomination of *Atacisaurus crassiproratus* (Middle Eocene; Issel, France) as cf. *Iberosuchus* (Crocodylomorpha: Metasuchia). Geobios 29:353–364.

Ortega, F., Z. Gasparini, A. D. Buscalioni, and J. O. Calvo. 2000. A new species of *Araripesuchus* (Crocodylomorpha, Mesoeucrocodylia) from the Lower Cretaceous of Patagonia (Argentina). Journal of Vertebrate Paleontology 20:57–76.

Ősi A. 2008. Cranial osteology of *Iharkutosuchus makadii*, a Late Cretaceous basal eusuchian crocodyliform from Hungary. *Neues Jahrbuch für Geologie und Paläontologie, Abhandlungen* 248:279–299.

Osmólska, H. 1972. Preliminary note on a crocodilian from the Upper Cretaceous of Mongolia.Palaeontologia Polonica 27:43–47.

Osmólska, H., S. Hua, and E. Buffetaut.1997.*Gobiosuchus kielanae* (Protosuchia) from the Late Cretaceous of Mongolia: anatomy and relationships. Acta PaleontologicPolonica 42:257–289.

Owen, R. 1878. Monograph on the fossil Reptilia of the Wealden and Purbeck Formations. Supplement VIII, Crocodilia (*Goniopholis*, *Petrosuchus*, and *Suchosaurus*). Palaeontographical Society Monographs 32(Number 149):1–15.

Owen, R. 1879. Monograph on the fossil Reptilia of the Wealden and Purbeck Formations. Supplement IX, Crocodilia (*Goniopholis*, *Brachydectes*, *Nannosuchus*, *Theriosuchus*, and *Nuthetes*). Palaeontographical Society Monographs 33(Number 155):1–19.

Paolillo, A., and O. J. Linares. 2007. Nuevos cocodrilos Sebecosuchia del Cenozoico suramericano (Mesosuchia: Crocodylia). Paleobiologia Neotropical 03:1–25.

Peng, G.-Z. 1995. A Late Jurassic protosuchian Sichuanosuchus huidongensis from Zigong, Sichuan Province. Vertebrata Palasiatica 34: 269-278.

Peng, G.-Z., and Shu, C.-K. 2005. A new species of *Hsisosuchus* from the Late Jurassic of Zigong, Sichuan, China. Vertebrata Palasiatica 43:312–324.

Pinheiro, A. E. P., R. J. Bertini, M. B. Andrade, and R. G. M. Neto. 2008. A new specimen of *Stratiotosuchus maxhechti* (Baurusuchidae, Crocodyliformes) from Adamantina Formation (Upper Cretaceous), Southeastern Brazil.Revista Brasileira de Paleontologia 11(1):37–50.

Pol, D. 1999a. El esqueleto postcraneano de *Notosuchus terrestris* (Archosauria: Crocodyliformes) del Cretácico Superior de la Cuenca Neuquina y su información filogenética. Licenciatura thesis, Facultad de Ciencias Exactas y Naturales, Universidad de Buenos Aires, 158 pp.

Pol, D.1999b. Basal mesoeucrocodylian relationships: new clues to old conflicts. Journal of Vertebrate Paleontology 19(3, Suppl.):69A.

Pol, D. 2003. New remains of *Sphagesaurus huenei* (Crocodylomorpha: Mesoeucrocodylia) from the Late Cretaceous of Brazil.Journal of Vertebrate Paleontology 23:817–831.

Pol, D., and S. Apesteguía. 2005. New *Araripesuchus* remains from the Early Late Cretaceous (Cenomanian) of Patagonia. American Museum Novitates 3490:1–38.

Pol, D., and I. H. Escapa. 2009. Unstable taxa in cladistics analysis: identification and the assessment of relevant characters. Cladistics 25:1–13.

Pol, D., and Z. Gasparini. 2009. Skull Anatomy of *Dakosaurus andiniensis* (Thalattosuchia: Crocodylomorpha) and the phylogentic position of Thalattosuchia. Journal of Systematic Palaeontology 7:165–197.

Pol, D., and M. A. Norell. 2004a. A new crocodyliform from Zos Canyon Mongolia.American Museum Novitates 3445:1–36.

Pol, D., and M. A. Norell. 2004b. A new gobiosuchid crocodyliform taxon from the Cretaceous of Mongolia.American Museum Novitates 3458:1–31.

Pol, D., and J. E. Powell 2011. A new basal mesoeucrocodylian from the Río Loro Formation (Paleocene) of northwestern Argentina.Zoological Journal of the Linnean Society 163:S7–S36.

Pol, D., A. H. Turner, and M. A. Norell. 2009. Morphology of the Late Cretaceous crocodylomorph *Shamosuchus djadochtaensis* and a discussion of neosuchian phylogeny as related to the origin of Eusuchia. Bulletin of American Museum of Natural History 324:1–103.

Pol, D., S.-H. Ji, J. M. Clark, and L. M. Chiappe. 2004. Basal crocodyliforms from the Early Cretaceous Tugulu Group (Xinjiang, China), and the phylogeneticposition of *Edentosuchus*. Cretaceous Research 25:603–622.

Price, L. I. 1945. A new reptile from the Cretaceous of Brazil.Notas Preliminares e Estudos, Servicio Geologia Mineralogia do Brasil 25:1–8.

Price, L. I. 1950.On a new crocodilian, *Sphagesaurus*, from the Cretaceous of the State of São Paulo, Brazil.Anais Academia Brasileira de Ciencas 22:77–83.

Price, L. I. 1955. Novos crocodilideos dos Arenitos da Série Bauru, Cretáceo do estado de Minas Gerais. Anais Academia Brasileira de Ciencias 27:487–498.

Price, L. I. 1959. Sobre um crocodilideo notossuquio do Cretacico Brasileiro. Boletim Divisão de Geolgia e Mineralogia Rio de Janeiro 118:1–55.

Puértolas-Pascual E, Canudo JI, Moreno-Azanza M. 2013. The eusuchian crocodylomorph *Allodaposuchus* *subjuniperus* sp. nov., a new species from the latest Cretaceous (upper Maastrichtian) of Spain. *Historical Biology*, 26:91–109.

Riff, D. 2003. Descrição morfológica do crânio e mandíbula de *Stratiotosuchus maxhechti* (Crocodylomorpha, Cretáceo Superior do Brasil) e seu posicionamento filogenético. M.Sc. thesis, Universidade Federal do Rio de Janeiro, 133 pp.

Riff, D. 2007. Anatomia apendicular de *Stratiotosuchus maxhechti* (Baurusuchidae, Cretáceo Superior do Brasil) e análise filogenética dos Mesoeucrocodylia. PhD dissertation, Universidade Federal do Rio de Janeiro, 395 pp.

Riff, D., and Kellner, A. W. A. 2011. Baurusuchid crocodyliforms as theropod mimics: clues from the skull and appendicular morphology of *Stratiotosuchus maxhechti* (Upper Cretaceous of Brazil). Zoological Journal of the Linnean Society 163:S37–S56.

Romer, A. S. 1923. Crocodilian pelvic muscles and their avian and reptilian homologues. Bulletin of the American Museum of Natural History 48:533–551.

Romer, A. S. 1972. The Chañares (Argentina) Triassic reptile fauna. XIII. An early ornithosuchid pseudosuchian, *Gracilisuchus stipanicicorum*, gen. et sp. nov. Breviora 389:1–24.

Rossmann, T., M. Rauhe, and F. Ortega. 2000. Studies on Cenozoic crocodiles 5. Description of *Bergisuchus dietrichbergi* Kühn (Sebecosuchia: Bergisuchidae n. fam.) from the Middle Eocene of Germany, and some new systematic and biological conclusions.Paläontologische Zeitschrift 74:379–392.

Rusconi, C. 1933. Sobre reptiles cretaceos del Uruguay (*Uruguaysuchus aznarezi*, n. g. n. sp) y sus relaciones con los notosúquidos de Patagonia. Boletín Instituto de Geología y Perforaciones Montevideo Uruguay 19:1–64.

Salisbury, S. W. 2002. Crocodilians from the Lower Cretaceous (Berriasian) Purbeck Limestone Group, southern England; pp. 121–144 in A. R. Milner and D. J. Batten (eds.), Life and environments in Purbeck times. Special Papers in Palaeontology, 68.

Salisbury, S. W., P. M. A. Willis, S. Peitz, and P. M. Sander. 1999. The crocodilian *Goniopholis simus* from the Lower Cretaceous of north-western Germany; pp. 121–148 in D. Unwin (ed.), Cretaceous Fossil Vertebrates. Special Papers in Palaeontology, 60.

Salisbury SW, Molnar RE, Frey E, Willis PMA. 2006. The origin of modern crocodyliforms: new evidence from the Cretaceous of Australia. *Proceedings of the Royal Society, B* 273:2439–2448.

Sereno, P. C., H. C. E. Larsson, C. A. Sidor, and B. Gado. 2001. The giant crocodyliform *Sarcosuchus* from the Cretaceous of Africa. Science 294:1516–1519.

Sereno, P.C., and Larsson, H. C. E. 2009. Cretaceous Crocodyliforms from the Sahara. ZooKeys 28:1-143.

Sertich, J. J. W., and J. R. Groenke. 2010. Appendicular Skeleton of *Simosuchus clarki* (Crocodyliformes: Notosuchia) from the Late Cretaceous of Madagascar. Society of Vertebrate Paleontology Memoir 10:122–153.

Stromer, E. 1914. Ergebnisse der Forschungsreisen Prof. E. Stromers in den Wüsten Ägyptens. II Wirbeltier-Reste der Baharîje-Stufe (unterstes Cenoman). 1. Einleitung und 2. *Libycosuchus*. Abhandlungen der Königlich Bayerischen Akademie der Wissenschaften 27:1–16.

Schwarz D. 2002. A new species of *Goniopholis* from The Upper Jurassic of Portugal. Palaeontology 45:185–208.

Schwarz D., Salisbury SW. 2005. A new species of *Theriosuchus* (Atoposauridae, Crocodylomorpha) from the Late Jurassic (Kimmeridgian) of Guimarota, Portugal. Geobios 38:779–802.

Troxell, E. L. 1925. *Hyposaurus*, a marine crocodilian.American Journal of Science 8:489– 514.

Turner, A. H. 2006. Osteology and phylogeny of a new species of *Araripesuchus* (Crocodyliformes: Mesoeucrocodylia) from the Late Cretaceous of Madagascar. Historical Biology 18:255–369.

Turner, A. H., and G. A. Buckley. 2008. *Mahajangasuchus insignis* (Crocodyliformes: Mesoeucrocodylia) cranial anatomy and new data on the eusuchian–style palate. Journal of Vertebrate Paleontology 28:382–408.

Turner, A. H., and J. O. Calvo. 2005. A new sebecosuchian crocodyliform from the Late Cretaceous of Patagonia. Journal of Vertebrate Paleontology 25:87–98.

Turner, A.H, and Sertich, J. J. W. 2010. Phylogenetic history of *Simosuchus clarki* (Crocodyliformes: Notosuchia) from the Late Cretaceous of Madagascar. Journal of Vertebrate Paleontology, Memoir 10:177-236.

Turner AH. In press. A review of *Shamosuchus* and *Paralligator* (Crocodyliformes, Neosuchia) from the Cretaceous of Asia. PloS One.

Tykoski, R. S., T. B. Rowe, R. A. Ketcham, and M. W. Colbert. 2002. *Calsoyasuchus valliceps*, a new crocodyliform from the Early Jurassic Kayenta Formation of Arizona. Journal of Vertebrate Paleontology 22:593–611.

Vignaud, P., and Z. Gasparini. 1996. New *Dakosaurus* (Crocodylomorpha, Thalattosuchia) in the Upper Jurassic of Argentina. Comptes Rendus de l'Académie de Sciences Paris, Sciences de la Terre 322:245–250.

Wellnhofer, P. 1971. Die Atoposauridae (Crocodylia, Mesosuchia) der Oberjura- Plattenkalke Bayerns. Palaeontographica, Abteilung A 138:133–165.

Wenz, S. 1968. Contribution a l ́étude du genere *Metriorhynchus*: crâne et moulage endocranien de *Metriorhynchus superciliosus*. Annals de Paléontologie 54:148–191.

Westphal, F. 1962. Die Krokodilier des deutschen und englischen oberen Lias. Palaeontographica, Abteilung A 118:23–118.

Wilkinson, M. W., Thorley, J. L. and Upchurch, P. M. 2000. A chain is no stronger than its weakest link: Double decay analyses of phylogenetic hypotheses. Systematic Biology 49:754-776.

Wilson, J. A., M. S. Malkani, and P. D. Gingerich. 2001. New crocodyliform (Reptilia, Mesoeucrocodylia) from the Upper Cretaceous Pab Formation of Vitakri, Balochistan (Pakistan). Contributions from the Museum of Paleontology University of Michigan 30:321– 336.

Woodward, A. S. 1896. On two Mesozoic crocodilians (*Notosuchus* genus novum and *Cynodontosuchus* genus novum) from the red sandstones of the territory of Neuquén.Annals Museo de La Plata (Palaeontologia Argentina) 4:1–20.

Wu, X.-C., and S. Chatterjee. 1993. *Dibothrosuchus elaphros*, a crocodylomorph from the Lower Jurassic of China and the phylogeny of the Sphenosuchia. Journal of Vertebrate Paleontology 13:58–89.

Wu, X.-C., and H.-D. Sues. 1996. Anatomy and phylogenetic relationships of *Chimaeresuchus paradoxus*, an unusual crocodyliform reptile from the Lower Cretaceous of Hubei, China. Journal of Vertebrate Paleontology 16:688–702.

Wu, X. -C., D. B. Brinkman, and J.C. Lu. 1994a. A new species of *Shantungosuchus* from the Lower Cretaceous of Inner Mongolia (China), with comments on *S. chuhsienensis* Young, 1961 and the phylogenetic position of the genus. Journal of Vertebrate Paleontology 14:210–229.

Wu, X.-C., D. B. Brinkman, and A. P. Russell. 1996. *Sunosuchus junggarensis* sp. nov. (Archosauria: Crocodyliformes) from the Upper Jurassic of Xinjiang, People‘s Republic of China. Canadian Journal of Earth Sciences 33:606–630.

Wu, X.-C., Z.-W. Cheng, and A. P. Russell. 2001b. Cranial anatomy of a new crocodyliform (Archosauria: Crocodylomorpha) from the Lower Cretaceous of Song-Liao Plain, northeastern China. Canadian Journal of Earth Sciences 38:1653–1663.

Wu, X.-C., J. Li, and X. Li. 1994b. Phylogenetic relationship of *Hsisosuchus*.Vertebrata PalAsiatica 32:166–180.

Wu, X.-C., A. P. Russell, and D. B. Brinkman. 2001a. A review of *Leidyosuchus canadensis* Lambe, 1907 (Archosauria: Crocodyliformes) and an assessment of cranial variation based upon new material. Canadian Journal of Earth Sciences 38:1665–1687.

Wu, X.-C., A. P. Russell, and S. L. Cumbaa. 2001c. *Terminonaris* (Archosauria: Crocodyliformes): new material from Saskatchewan, Canada, and comments on its phylogenetics relationships. Journal of Vertebrate Paleontology 21:492–514.

Wu, X.-C., H.-D. Sues, and Z.-M. Dong. 1997. *Sichuanosuchus shuhanensis*: a new ?Early Cretaceous protosuchian (Archosauria: Crocodyliformes) from Sichuan (China), and the monophyly of Protosuchia. Journal of Vertebrate Paleontology 17:89–103.

Young, C. C. 1973. A new fossil crocodile from Wuerho. Memoirs of the Institute of Vertebrate Paleontology and Paleoanthropology 11:37–44. [In Chinese].

Young, C. C., and M. C. Chow. 1953. New fossil reptiles from Szechuan China. Acta Paleontologica Sinica 1:1–87.

Zaher, H., D. Pol, A. B. Carvalho, C. Riccomini, D. Campos, and W. Nava. 2006. Redescription of the cranial morphology of *Mariliasuchus amarali*, and its phylogenetic affinities (Crocodyliformes, Notosuchia). American Museum Novitates 3512:1–40.
